# White-Light Emitting Di-Ureasil Hybrids

**DOI:** 10.3390/ma11112246

**Published:** 2018-11-12

**Authors:** Ming Fang, Lianshe Fu, Rute A. S. Ferreira, Luís D. Carlos

**Affiliations:** Department of Physics, CICECO-Aveiro Institute of Materials, University of Aveiro, 3810-193 Aveiro, Portugal; mingfang@ua.pt (M.F.); rferreira@ua.pt (R.A.S.F.); lcarlos@ua.pt (L.D.C.)

**Keywords:** di-ureasil organic–inorganic hybrids, in situ sol–gel synthesis, white light emission, lanthanide complexes, coumarin 1

## Abstract

White-light emitting materials have emerged as important components for solid state lighting devices with high potential for the replacement of conventional light sources. Herein, amine-functionalized organic-inorganic di-ureasil hybrids consisting of a siliceous skeleton and oligopolyether chains codoped with lanthanide-based complexes, with Eu^3+^ and Tb^3+^ ions and 4,4′-oxybis(benzoic acid) and 1,10-phenanthroline ligands, and the coumarin 1 dye were synthesized by in situ sol–gel method. The resulting luminescent di-ureasils show red, green, and blue colors originated from the Eu^3+^, Tb^3+^, and C1 emissions, respectively. The emission colors can be modulated either by variation of the relative concentration between the emitting centers or by changing the excitation wavelength. White light emission is achieved under UV excitation with absolute quantum yields of 0.148 ± 0.015, 0.167 ± 0.017, and 0.202 ± 0.020 at 350, 332, and 305 nm excitation, respectively. The emission mechanism was investigated by photoluminescence and UV–visible absorption spectroscopy, revealing an efficient energy transfer from the organic ligands to the Ln^3+^ ions and the organic dye, whereas negligible interaction between the dopants is discerned. The obtained luminescent di-ureasils have potential for optoelectronic applications, such as in white-light emitting diodes.

## 1. Introduction

White-light emitting materials have recently gained considerable attention owing to their broad applications in solid state lighting and full-color displays [1,2]. Generally, the realization of white-light emission requires the generation and intensity control of three (blue, green, and red) or two (blue and yellow) primary colors covering the whole visible spectral range (400 to 700 nm). Trivalent lanthanide (Ln^3+^)-based complexes are excellent candidates for designing multi-color luminescent materials due to their high photoluminescence efficiency via organic ligand’s sensitization (the well-known ‘antenna effect’) and narrow *f*–*f* band emission characteristics [3,4,5,6]. Consequently, a wide range of colors—for instance red (Eu^3+^ and Sm^3+^), green (Tb^3+^), blue (Tm^3+^ and Ce^3+^), and yellow (Dy^3+^)—can be obtained by choosing different Ln^3+^–based complexes with suitable ligands. Up to now, Ln^3+^ ions have been incorporated into many organic, inorganic, and organic–inorganic hybrid hosts, such as organic soft gels [6,7], polymers [8,9,10,11], core–shell microspheres [12], metal–organic frameworks (MOFs) [13,14,15], and organosilica hybrids [16], to prepare white-light emitting materials. Three main synthesis strategies were adopted: (i) three-component approach; (ii) two-component approach, and (iii) one-component approach [17]. In the first case, three distinct Ln^3+^ ions were used to produce white-light emission [18,19,20,21,22,23,24], such as in Ln^3+^-based isostructural MOFs codoped with Eu^3+^/Tb^3+^/Gd^3+^ or Eu^3+^/Tb^3+^/La^3+^ that can generate white-light by fine-tuning the Ln^3+^ ions molar ratios. In these materials, the optically inactive ions (Gd^3+^ and La^3+^) are usually used as optical dilutors to augment the ligand’s blue emission from the organic ligands. In the (ii) two-component approach, two distinct Ln^3+^ ions coexist in a single phase of the white-light emitting materials [18,25,26,27,28,29]. For instance, in Eu^3+^-doped Gd^3+^ isostructural MOFs color-tunable luminescence and white-light emission are realized by varying the excitation wavelength [25]. Finally, in the (iii) one-component approach, white-light is attained combining the emission of a single Ln^3+^ ion with the complementary one of organic ligands or dyes [30,31]. An intriguing example is the combination of a Eu^3+^ complex with a naphthalimide derivative that generates white-light emission from the color balance of isolated naphthalimide molecules (blue), aggregated naphthalimide excimers (green) and Eu^3+^ ions (red) [31]. White light emission was also attained by mixing Eu^3+^ ions with an organic ligand containing a blue-emitting coumarin group and a green-emitting rhodamine 6G moiety [30].

Among these materials, MOFs of Ln^3+^-based complexes have been most frequently applied to generate white-light emission due to their structural diversities and stabilities. In these Ln^3+^-based MOFs, the organic ligands (e.g., polycarboxylate ligands) act both as linkers, for the formation of the framework, and as sensitizers, for the emission enhancement of the Ln^3+^ ions via the ‘antenna effect’. It should be noted that in these white-light emitting materials, the blue primary color source, is mainly originated either from the organic ligands or from the codoping with optically inactive ions, e.g., La^3+^ or Gd^3+^. In the former case, the blue emission is induced by the mismatch between the low-energy ligand triplet state and the excited levels of the Ln^3+^ ion, resulting in an inefficient ligand-to-metal energy transfer. In the latter case, however, the incorporation of higher amounts of optically inactive ions (such as La^3+^, Gd^3+^, or Lu^3+^) decreases the efficiency of the ligand-to-metal energy transfer suppressing, concomitantly, the red (Eu^3+^) and green (Tb^3+^) emissions and enhancing the ligand blue component. In both cases, the process to generate white-light is quite ineffective resulting in relatively low overall emission quantum yields. One of the strategies to address this problem is the synthesis of Dy^3+^-based MOFs emitting in the blue (^4^F_9/2_→^6^H_15/2_), yellow (^4^F_9/2_→^6^H_13/2_), and red (^4^F_9/2_→^6^H_11/2_) spectral regions, upon UV excitation. In some examples, this 4*f* emission is combined with those of the MOF organic counterpart [32,33,34]. Alternatively, white-light emission is also achieved with Dy^3+^ complexes, in solution and as solid powders [35]. By selecting a proper excitation wavelength, the relative intensity between the Dy^3+^ and the ligand emissions are balanced to yield white-light visible to the naked eye. In addition, as Ce^3+^ can also emits in the blue region the combination of well-chosen relative ratios of Ce^3+^, Tb^3+^, and Eu^3+^ led to white-light emission under UV excitation [36].

An alternative strategy to yield efficient blue emission is to introduce strong blue fluorescent molecules with green- and red-emitters into MOFs. Fluorescent dyes are a good choice as they show high emission quantum yields. When embedded into hosts, the dyes are isolated from each other preventing aggregation-induced quenching, which restricts internal molecular motions and suppresses nonradiative relaxations [37,38,39]. For example, the 7-diethylamino-4-methylcoumarin (C1) dye with blue emission was incorporated into Eu^3+^/Tb^3+^ MOFs and white-light emission with high quantum yield was obtained [37]. In other examples, white-light was generated combining the blue emission of an organic ligand and the yellow one of rhodamine B [39] or joining three distinct dyes and playing either with the amount or type of the dyes, or with the excitation wavelength [38].

**Scheme 1:**
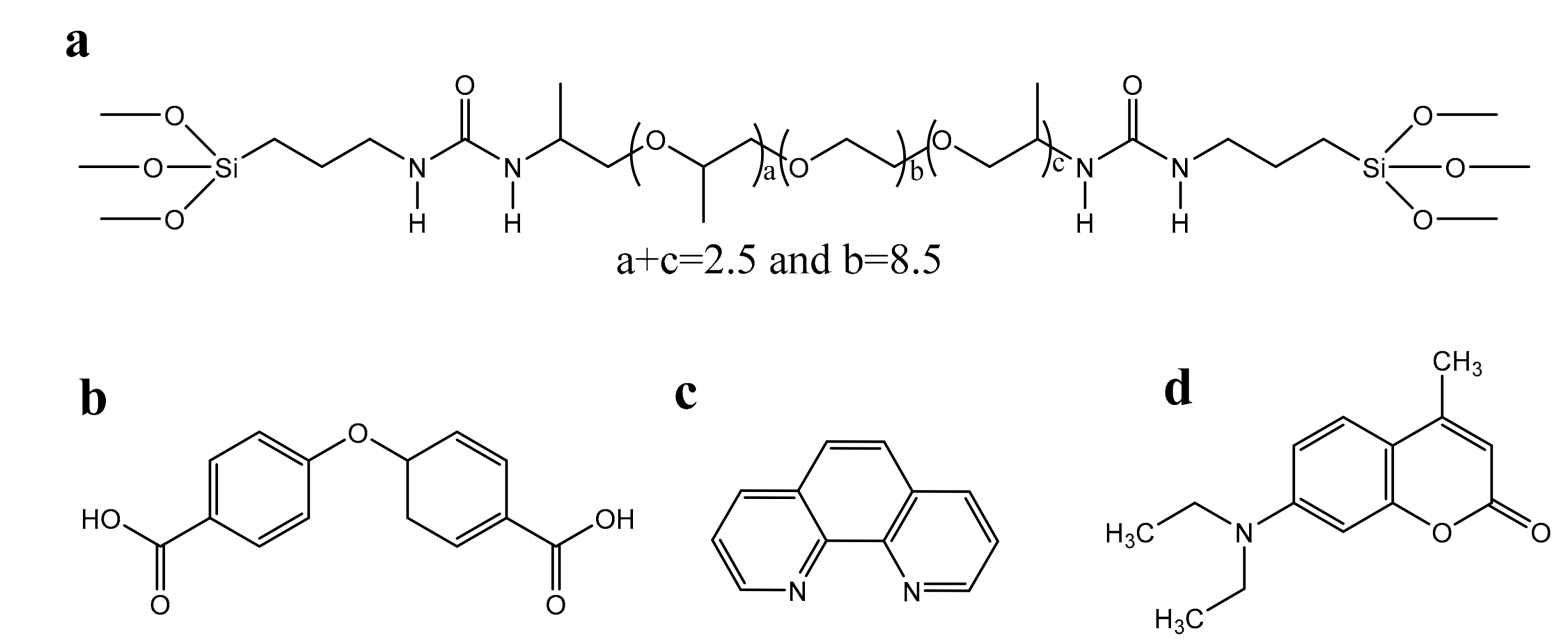
Molecular structures of (**a**) d-U(600), (**b**) 4,4′-oxybisbenzoic acid (Oba), (**c**) 1,10-phenanthroline (Phen) and (**d**) C1.

The above Ln^3+^-based white-light emitting materials are crystalline, which restrict their broad application due to the difficulty of processability as films. Moreover, from the optoelectronic application point of view, generally, the materials should have high transparency in the wavelength range of interest, low phonon energy, and good photostability, as well as processability for flexible electronics. Amine-functionalized d-U(600) di-ureasil is formed by poly(oxyethylene) chains cross-linked to a siloxane skeleton by urea “bridges” (Scheme 1) and are promising host materials for embedding optically active centers and generating white-light emission [40,41].

Although some efforts have been devoted to this field, the design and synthesis of white-light emitting materials with high emission quantum yields are still a challenging task. Indeed, different emissive moieties are needed to attain primary colors and their relative concentration ratio require a precise adjust for compensating and balancing luminescence colors and to modulate the energy transfer processes between them. Accordingly, the purpose of this work is to synthesize transparent di-ureasils with high emission quantum yield to generate white-light emission. Herein, we use 4,4′-oxybisbenzoic acid (Oba) and 1,10-phenanthroline (Phen) as organic ligands for sensitization of Eu^3+^ and Tb^3+^ emissions. The molecular structures of Oba and Phen are presented in Scheme 1. Oba, a typical flexible V-shaped dicarboxylate ligand [42], has been already employed to prepare Ln^3+^-based complexes in di-ureasils by in situ sol–gel synthesis and the resulting materials exhibit promising luminescence properties [43]. To boost the blue component for the white-light emitting di-ureasil, herein, the widely used organic dye C1 [44,45,46] (Scheme 1) was adopted. Moreover, since Ln^3+^-based complexes with Oba are usually not dissolved in common organic solvents in ambient environment and, thus, they are difficult to be incorporated directly into a certain host homogenously, the in situ sol–gel technique was applied. The luminescence properties of the doped di-ureasils and the achieved white-light emission are discussed and the energy transfer mechanisms addressed.

## 2. Materials and Methods

### 2.1. Materials and Synthesis

The details concerning the chemicals used are given out in Supplementary Material. All the di-ureasils were synthesized according to the procedure described in the Scheme S1 (Supplementary Material). The detailed synthesis procedure, as well as the adopted designations for all the luminescent hybrids (Table S1) are also provided in Supplementary Material. Single-doped di-ureasils with Eu^3+^, Tb^3+^ (in the presence of Oba and Phen as ligands), and C1 are denoted as Eu-I, Tb-I, are C1-I, respectively. Eu^3+^/Tb^3+^ codoped di-ureasils (with Oba and Phen ligands) are designated as EuTb-I, whreas the triply-doped hybrids with the same amount of Eu^3+^ and Tb^3+^ sensitized by Oba and Phen but with distinct C1 concentrations (low, medium, and high contents) are named as EuTbC1-I, EuTbC1-II, and EuTbC1-III, respectively.

### 2.2. Characterization

The powder X-ray diffraction (XRD) patterns were recorded in the 2θ range spanning from 3.5 to 60.0° by using a Ragaku-D/Max 2500 diffractometer system under exposure of Cu K_α_ radiation (1.54 Å) at room temperature. The Fourier-transform infrared (FT-IR) spectra were obtained by using a MATTSON 7000 FT-IR Spectrometer system to scan the sample absorbance intensity from 4000–400 cm^−1^ with 64 scans and a 4 cm^−1^ resolution. UV–visible (UV–vis) absorption spectra were measured in transparent thin films (about 10^−3^ m thickness) using a dual-beam spectrometer Lambda 950 (PerkinElmer) over the scan range 250–500 nm and a resolution of 1.0 nm. Thermogravimetric (TG) measurements were performed with a 10 °C/min heating speed under air atmosphere on SDT 2960 analyzer (Shimadzu, Japan). The excitation and emission spectra were recorded using a Fluorolog3^®^ Horiba Scientific Spectrofluorometer (Model FL3-2T) with a 450 W Xe arc lamp, a modular double grating excitation spectrometer, and a TRIAX 320 single emission monochromator coupled to an R928 Hamamatsu photomultiplier. The front face acquisition mode is used. The emission spectra were corrected for detection and optical spectral response of the spectrofluorimeter and the excitation spectra were corrected for the spectral distribution of the lamp intensity using a photodiode reference detector. The emission decay curves were measured with the setup described for the luminescence spectra using a pulsed Xe–Hg lamp (6 μs pulse at half width and 20–30 μs tail). Measurements at 12 K were performed on a helium-closed cycle cryostat with vacuum system measuring around 5 × 10^−9^ bar. The absolute emission quantum yields (q) were measured at room temperature using a quantum yield measurement system C13534 from Hamamatsu with a 150 W xenon lamp coupled to a monochromator for wavelength discrimination, an integrating sphere as sample chamber and multichannel analyzers for signal detection in the visible and in the NIR ranges. Three measurements were made for each sample so that the average value with an accuracy of 10% is reported. The white-light emission was further quantified by calculating the Commission Internationale de l’Eclairage (CIE) emission chromaticity coordinates (x, y) for the 2° observer. The colour correlated temperature (CCT) values can be calculated through the polynomial function: CCT = 449t^3^ + 3525t^2^ + 6823.3t + 5520.33 with t = (x − 0.3320)/(0.1858 − y) [47]. 

## 3. Results and Discussion

### 3.1. Structural Studies

After the in situ synthesis of Ln^3+^ complexes, the structural features of the resulting luminescent hybrids were studied by XRD, FT-IR spectra, and TG analysis. 

The XRD patterns of d-U(600), Eu-I, Tb-I, EuTb-I, EuTbC1-I, EuTbC1-II, and EuTbC1-III display the typical amorphous ordering of siliceous domains [48] with broad bands centered at 20.8 ± 0.1, 21.6 ± 0.1, 21.2 ± 0.1, 21.2 ± 0.1, 21.1 ± 0.1, 21.2 ± 0.1, and 21.0 ± 0.1°, respectively, (Figure S1, Supplementary Material). In addition, all the patterns have a shoulder around 11° and a weak second-order hump spanning from 40 to 50°, typical of d-U(600) [41]. According to Bragg law [49], the structural unit distances of d-U(600), Eu-I, Tb-I, EuTb-I, EuTbC1-II, and EuTbC1-III depend on the bands around 21° being 4.3 ± 0.1, 4.1 ± 0.1, 4.2 ± 0.1, 4.2 ± 0.1, 4.2 ± 0.1, 4.2 ± 0.1, and 4.2 ± 0.1 Å, respectively. This distance is larger than two times the distance of the Si–O (1.62 Å) bond [41], indicating that the siliceous domains are based on several near-neighbor Si–O–Si structures. 

The FT-IR spectra of d-U(600), Eu-I, Tb-I, EuTb-I, EuTbC1-I, EuTbC1-II, and EuTbC1-III were investigated in the range 2000–500 cm^−1^, as presented in Figure S2 (Supplementary Material). The characteristic “amide I” (1800–1600 cm^−1^) and “amide II” regions (1600–1500 cm^−1^) of the “urea bridges” [50,51,52] are readily distinguished in the spectra. The “amide I” region mainly consists of three component signals from C=O stretching, CN stretching and C–C–N deformation absorptions, whereas for “amide II” the signals are ascribed to N–H in-plane bending, C–N stretching, and C–C stretching vibrations [50,51]. The signal located at 1713 cm^−1^ is used as an evidence of the formation of less ordered urea–polyether hydrogen-bonded associations induced by the Ln^3+^ coordination to the oxygen atom of the C=O group [43]. Here, the increased vibration intensity of the shoulder at 1713 cm^−1^ after the addition of Eu^3+^/Tb^3+^ ions evidences the interaction between the metal ions and the urea bridges through the coordination to the C=O group. Meanwhile, for all the spectra the most intense vibration bands around 1108 cm^−1^, with two shoulders at 1190 and 1130 cm^−1^, are attributed to the overlap of C–O stretching with the Si–O–Si and Si–O–C vibrations [53]. In addition, the vibration band appeared at around 920 cm^−1^ is assigned to the SiO–H bending vibration, due to the incomplete condensation reaction of the precursor during the sol–gel process [24]. 

The thermal decomposition processes were analyzed under air atmosphere environment, (Figure S3, Supplementary Material). The d-U(600) starts to decompose at 175 °C losing weight through two main stages. From 175 to 327 °C, d-U(600) displays a fast decomposition process with about 53.0% of weight loss. After further raising the temperature up to 600 °C, the total weight changes from 47.0% to about 16.0% ending with 14.4% at 800 °C, which is consistent with the theoretical value (13.8%) of d-U(600) decomposition with complete hydrolysis and condensation. The decomposition profiles of the doped hybrids are very similar to that of the undoped d-U(600), except fora slightly attenuation from 175 to 152 °C. When temperature was raised to 175 °C, all the doped samples have around 0.5 to 2.5% weight losses, which are ascribed to the solvents and/or water leakage. The decomposition of the organic ligands may happens from 152 to 327 °C, accordingly to the reported data on [(Eu(btfa)_3_(MeOH)_2_)⋅bpeta] and [Eu(tta)_3_(H_2_O)_2_] (where btfa is the 4,4,4-trifluoro-1-phenyl-1,3-butanedionate ion, bpeta is 1,2-bis(4-pyridyl)ethane, and tta– is 2-thenoyltrifluoracetonate) [54,55]. The total weight changes at 800 °C, 16.4, 13.8, 17.5, 15.6, 14.6, and 15.4%, for Eu-I, Tb-I, EuTb-I, EuTbC1-I, EuTbC1-II, and EuTbC1-III, respectively, are in agreement with the theoretical value (14.2%) considering the calculations of the decomposition of d-U(600) and lanthanide complexes. 

### 3.2. Optical Properties

**Figure 1:**
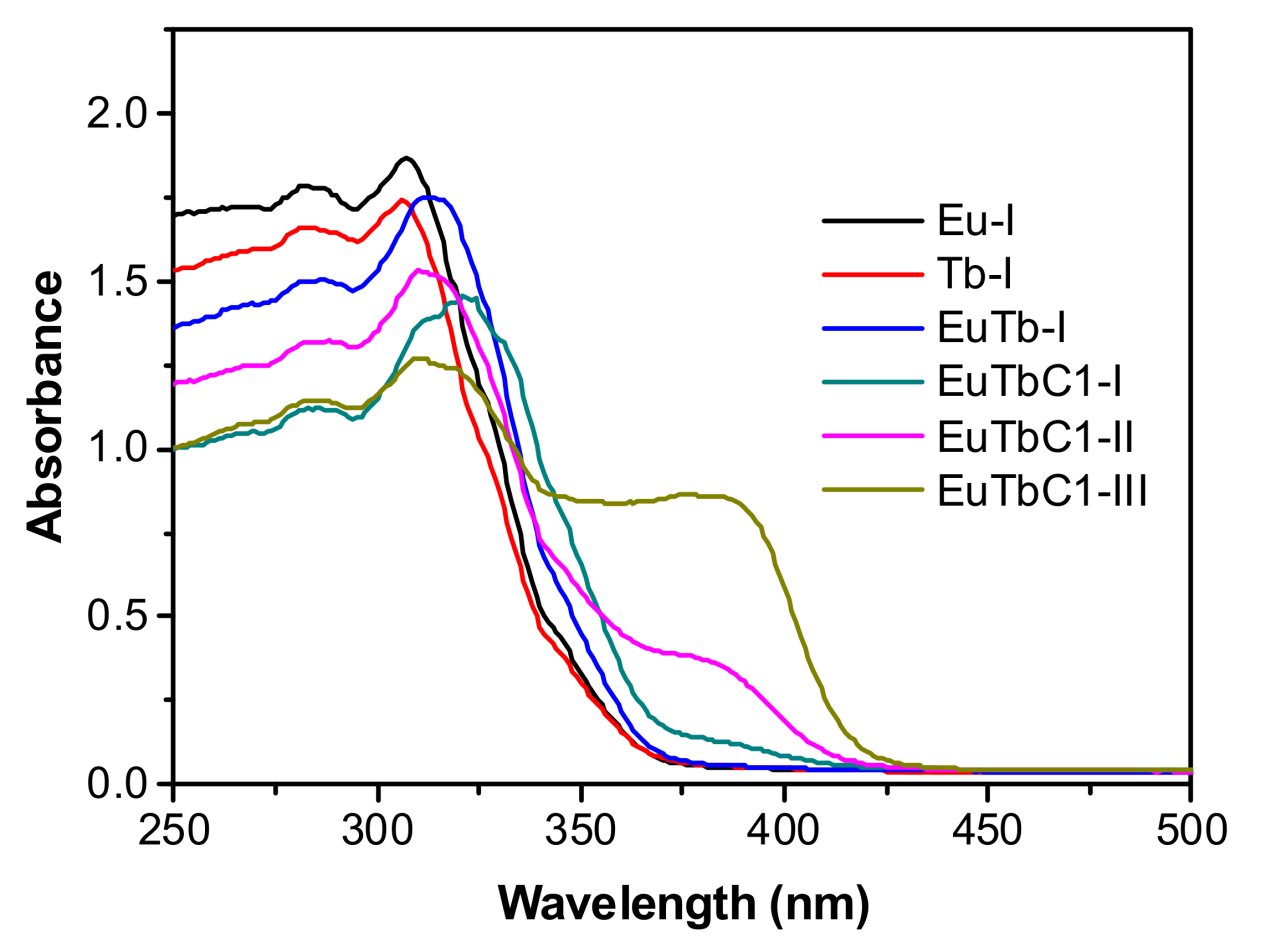
UV–vis spectra of Eu-I, Tb-I, EuTb-I, EuTbC1-I, EuTbC1-II, and EuTbC1-III.

The UV–vis spectra of the transparent thin films of Eu-I, Tb-I, EuTb-I, EuTbC1-I, EuTbC1-II, and EuTbC1-III are shown in Figure 1. For Eu-I, Tb-I, and EuTb-I, the high-energy peaks at around 285 and 310 nm are attributed mainly to the π → π*/n → π* transitions of Oba and Phen [43,56]. For EuTbC1-I, besides those high-energy peaks, a weak absorption band appeared at around 380 nm and is ascribed to the π → π* transition of C1 [57]. The absorption spectra of EuTbC1-I, EuTbC1-II, and EuTbC1-III show a pronouncedly increased absorbance in the 340–425 nm range with increasing C1 concentration which is attributed through density functional theory (DFT) and time-dependent DFT (TD-DFT) to the π → π* transition (with charge transfer characteristics), as shown in Figures S4 and S5 (Supplementary Material). 

The excitation spectra of Eu-I and Tb-I were measured by monitoring the more intense emission peaks of Eu^3+^ (at 615 nm) and Tb^3+^ (at 545 nm), Figure S6a, Supplementary Material. All the spectra display a broad band in the UV region until around 400 nm. The higher energy bands below 300 nm are related to the π → π* transitions of the organic ligands [58], whereas the lower energy bands are ascribed to the absorptions from both d-U(600) and the ligands. No *f*–*f* transitions from Eu^3+^ and Tb^3+^ ions were observed, except a small peak appeared for Eu-I and ascribed to the ^7^F_0_ → ^5^L_6_ transition, indicating a high-efficient ligand’s sensitization [59].

**Figure 2:**
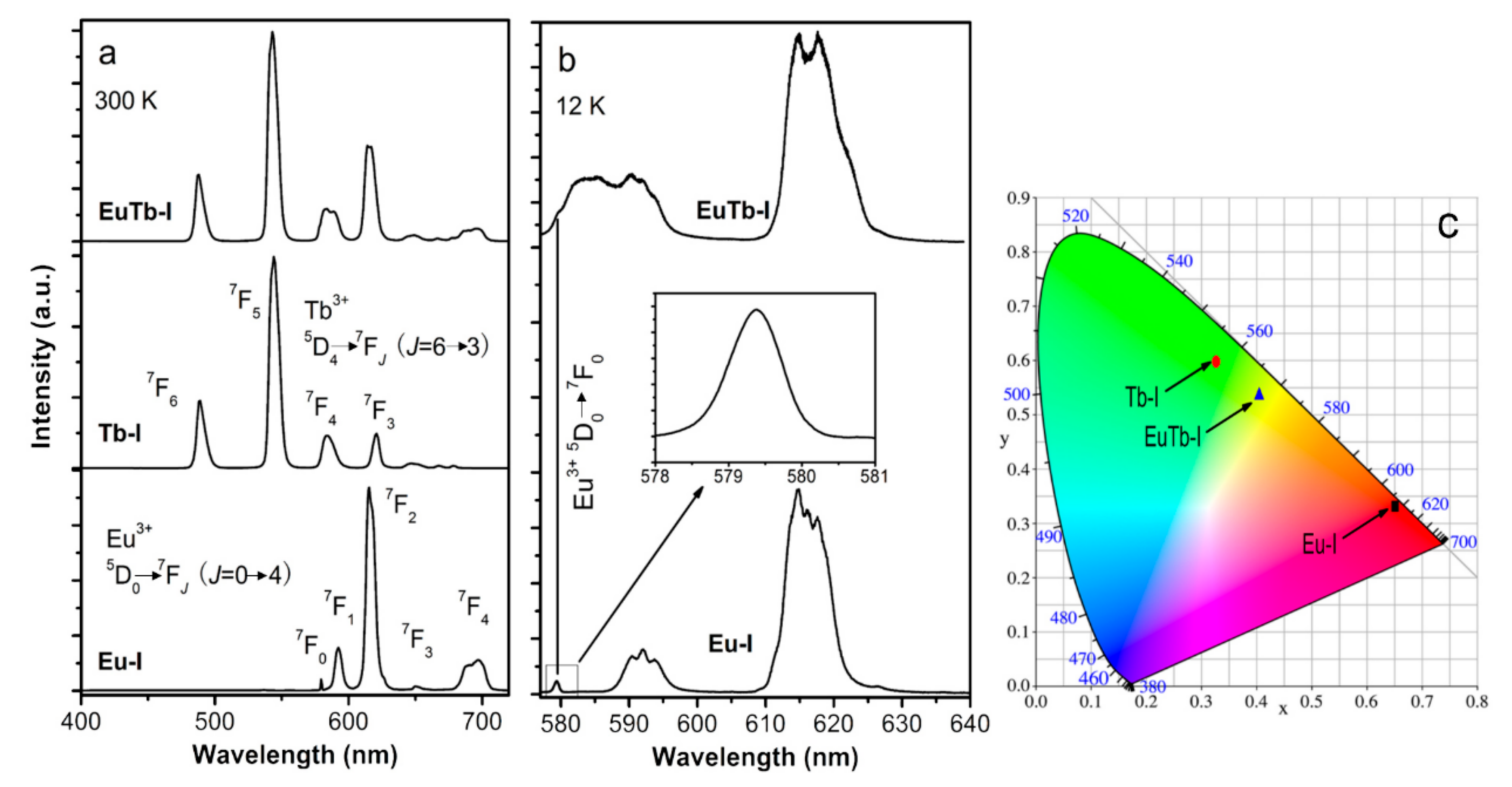
(**a**) Emission spectra (300 K) of Eu-I, Tb-I, and EuTb-I; (**b**) high-resolution emission spectra (12 K) of Eu-I and EuTb-I (excited at 295 nm); and (**c**) CIE chromaticity color diagram showing the hybrids’ corresponding emission color coordinates (300 K).

Upon excitation at 295 nm, Eu-I and Tb-I exhibit the typical emission spectra characteristic of the ^5^D_0_ → ^7^F_0-4_ (Eu^3+^) and ^5^D_4_ → ^7^F_6-3_ (Tb^3+^) transitions, Figure 2a. The chromaticity coordinates of Eu-I and Tb-I under 295 nm are in the red (0.65, 0.33) and green (0.33, 0.60) spectral regions, respectively, Figure 2c. For Eu-I, the unequivocal single component of the nondegenerate ^5^D_0_ → ^7^F_0_ transition points out a unique Eu^3+^ local environment (Figure 2b and Figure S6b in Supplementary Material). Moreover, the *J*-degeneracy splitting of the ^7^F_1-2_ levels in three and five components, respectively, indicate that the single Eu^3+^ local site has low symmetry without an inversion center. For Eu-I and Tb-I, and under excitation within the organic ligands (below 300 nm), the emission components of the ligands and of the d-U(600) in the blue region are absent, suggesting an efficient ligand/host-to-metal ion [60,61]. However, it is interesting to notice that relative strong Eu^3+^ and Tb^3+^ emissions are still detected under 350 nm irradiation (Figure S6b,c in Supplementary Material), demonstrating the potential application of these hybrids for white-light applications. 

**Figure 3:**
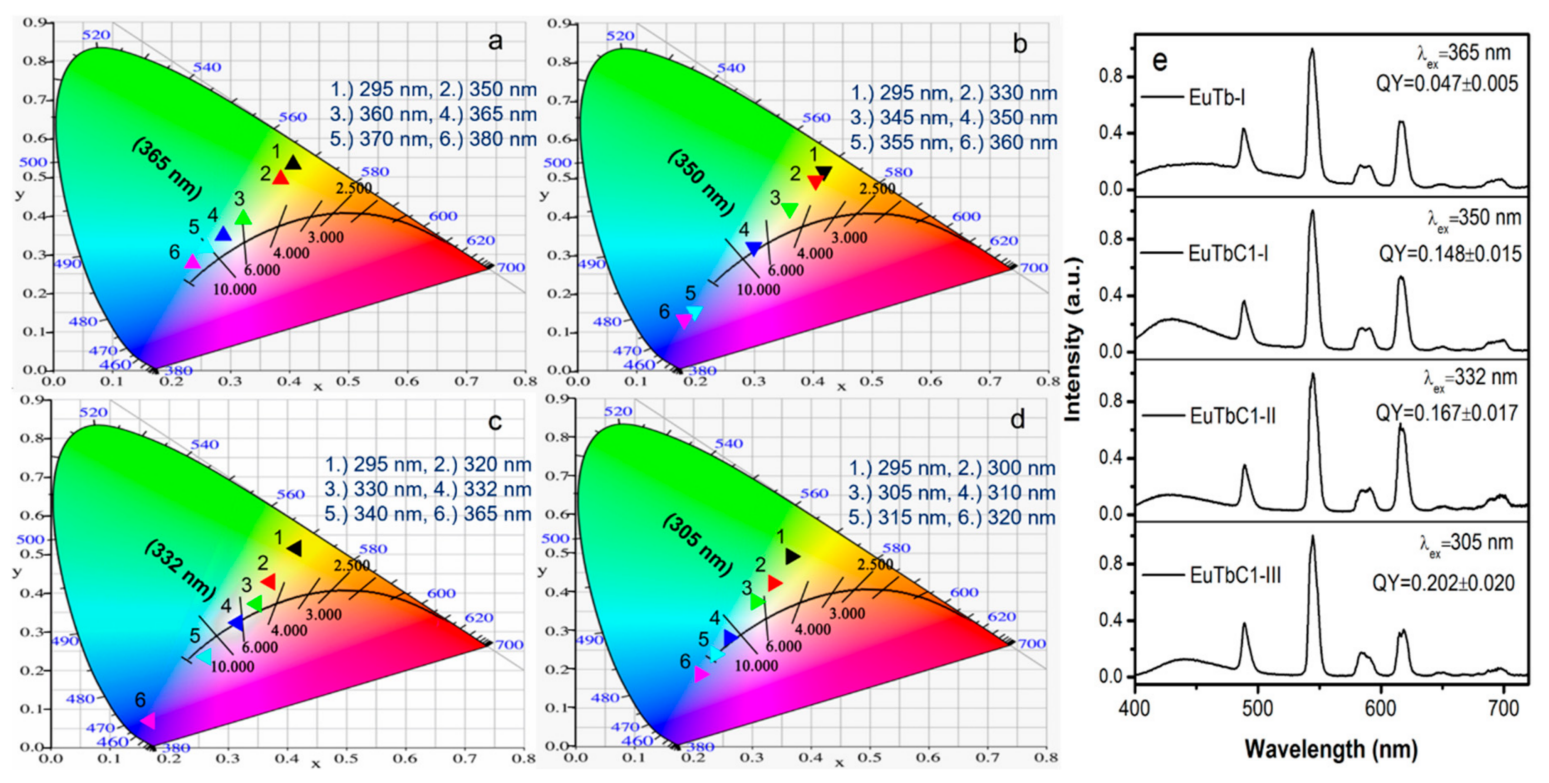
(**a**–**d**) CIE chromaticity diagrams showing the emission color coordinates of EuTb-I, EuTbC1-I, EuTbC1-II, and EuTbC1-III, respectively; and (**e**) the corresponding emission spectra.

**Figure 4:**
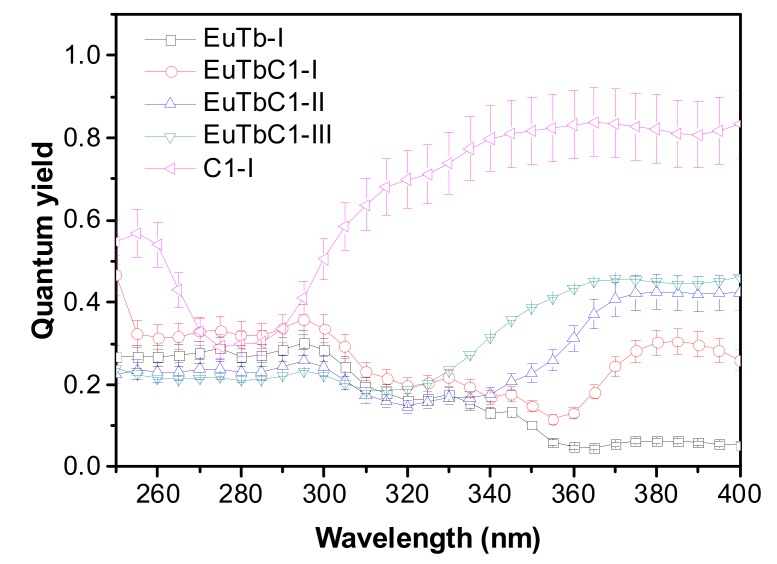
Wavelength dependence of the emission quantum yield values of EuTb-I, EuTbC1-I, EuTbC1-II, EuTbC1-III, and C1-I.

Figure 2a shows that yellow emission with color coordinates of (0.405,0.535), Figure 2c, can be realized for EuTb-I under 295 nm irradiation due to the contribution of the Eu^3+^ and Tb^3+^ emissions. The high-resolution emission spectrum measured at 12 K displays a *J*-degeneracy splitting similar than that observed for Eu-I, despite the superposition of Tb^3+^ emissions, Figure 2b. The emission spectra of EuTb-I with different excitation wavelengths and the corresponding CIE chromaticity coordinates are given in Figure S7 (Supplementary Material) and Figure 3a, respectively. When excited at higher energy wavelengths, EuTb-I shows the intense Eu^3+^ and Tb^3+^ emissions. With increasing excitation wavelength, the blue emission is gradually augmented and emission color in the white (excited at 365 nm) and cyan (excited at 380 nm) spectral regions is obtained. In Figure 3e, the emission spectrum excited at 365 nm gives out the blue emission centered at 460 nm, together with the Eu^3+^ and Tb^3+^ emissions, resulting in an overall white-light emission with chromaticity coordinates (0.287,0.351) and CCT = 7650 K. The corresponding excitation spectra monitored at 460, 544, and 615 nm display distinct π → π* transitions (Figure S7b, Supplementary Material). The absence of Tb^3+^ lines while monitoring within the Eu^3+^ emission indicates that no Tb^3+^-to-Eu^3+^ energy transfer occurs. Furthermore, the significant attenuation of the Eu^3+^ and Tb^3+^ emission intensities when the excitation wavelength is changed in the 360–380 nm range, results in low quantum yield values for the white-light emission (Figure 4), as it will be discussed later. 

To overcome the low quantum yield values of the codoped di-ureasils displaying white-light emission, C1 was incorporated into d-U(600) as the primary blue emission component. Herein different doping contents were used. For EuTbC1-I, and under 350 nm irradiation (Figure 3e), the blue color relative intensity (mainly spanning 400–480 nm) is obviously enhanced compare to the emission spectrum of EuTb-1. According to the excitation spectra (Figure S8b, Supplementary Material) and UV–vis spectra (Figure 1), this blue emission is mainly ascribed to the C1 dye. Changing the excitation wavelength, the relative components of each primary color (red, green, and blue) can be modulated (Figure S8a, Supplementary Material). When the excitation wavelength is 350 nm, EuTbC1-I displays blue, green and red emission bands and the overall color falls most closely to the ideal white-light point, with CIE chromaticity coordinates of (0.299,0.323) and CCT = 7372 K (Figure 3b).

Increasing the C1 content in EuTbC1-II and EuTbC1-III increases the blue color emission, as indicated in Figures S9 and S10 (Supplementary Material) and Figure 3c,d. The blue component does not display any shift with the variation of the excitation wavelength, Figure S7a (Supplementary Material), permitting to unequivocally assign it to the C1 dye. In fact, the blue emission of the d-U(600) host displays a typical red-shift with the increase of the excitation wavelength due to the nature of the two types of emitting centers (NH/C=O group of the urea cross-linkages and siliceous nanoclusters interface [62,63]). Consequently, white-light emission can be obtained exciting EuTbC1-II and EuTbC1-III at 332 and 305 nm with CIE chromaticity coordinates of (0.314, 0.324) and (0.307, 0.373), as well as the corresponding CCT = 6470 and 6495 K, respectively.

Figure 4 shows the absolute emission quantum yield values of EuTb-I, EuTbC1-I, EuTbC1-II, EuTbC1-III, and C1-I measured for excitations wavelengths between 250 and 400 nm. For excitations wavelengths up to 330 nm, all the hybrids present a similar wavelength dependence with values around 0.30 ± 0.03, for EuTb-I and EuTbC1-I, and 0.22 ± 0.02, for EuTbC1-II and EuTbC1-III. For these excitation wavelengths the emission is dominated by the Eu^3+^ and Tb^3+^ transitions. The decrease in the quantum yield values with the increasing of C1 concentration (as also observed for the absorbance, Figure 1) suggests the occurrence of extra non-radiative mechanisms related to C1. For higher excitation wavelengths (>330 nm), however, the emission quantum yields increase with the increasing of the C1 amount in the hybrid (the absorbance also increases with C1 concentration in that region, Figure 1). This is explained because, at these longer wavelengths, the hybrids’ emission is dominated by the C1 component that presents a very high quantum yield, around 0.80 ± 0.08 (the Eu^3+^ and Tb^3+^ emissions are negligible, Figures S8–S10, Supplementary Material). Comparing to other Ln^3+^-doped white-light emitting materials, EuTbC1-III displays a higher quantum yield value due to the combination of Eu^3+^ and Tb^3+^ emissions with that of C1, Table S2 (Supplementary Material).

**Figure 5:**
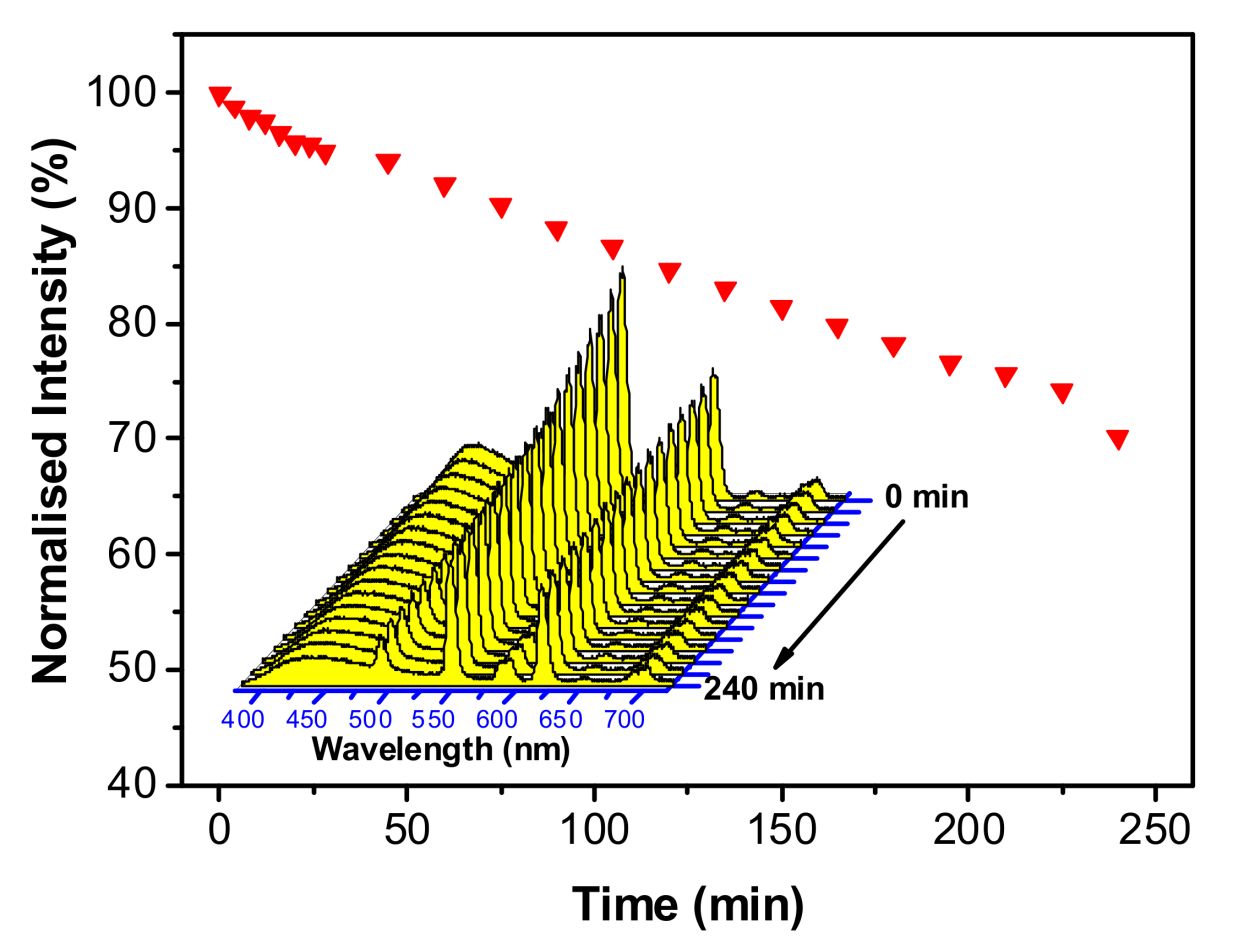
Photostability of EuTbC1-I irradiated at 350 nm.

The photo-stability of EuTbC1-I was evaluated by measuring the emission with continuous irradiation during 4 h, Figure 5. The overall emission intensity, calculated integrating the areas of the emission bands between 380 and 720 nm, drops around 30% in that period. Furthermore, the chromaticity coordinates of the hybrid changes from (0.299, 0.318) to (0.316, 0.332), as seen in Figure S11 (Supplementary Material).

To gain insights on the mechanism of the whit-light emission, the Ln^3+^ ligand sensitization and the energy transfer processes were studied. Figure S12 (Supplementary Material) displays the normalized excitation spectra of EuTb-I, EuTbC1-I, EuTbC1-II, and EuTbC1-III (monitoring the Eu^3+^ and Tb^3+^ emissions). All the spectra are similarly being dominated by the π → π* transitions of the organic ligands (<300 nm) and by the excited states of both d-U(600) and the ligands (300–350 nm, as already noticed, Figure 1). The absence of the typical C1 absorption at longer wavelengths (350–420 nm, Figure S13 in Supplementary Material) points out that the Tb^3+^ and Eu^3+^ excited states are not populated through energy transfer arising from C1 levels. The UV–vis and emission spectra of C1 in EtOH (1.0 × 10^−5^ M) are shown in Figure S13 (Supplementary Material), which are used to estimate the excitation absorption centering at 27,000 cm^−1^ and S_1_ state of C1 around 23,000 cm^−1^.

The Oba and Phen ligands dominate the Eu^3+^ and Tb^3+^ excitation. The energy transfer pathway for the metal ion sensitization involves the excitation of the excited organic ligand singlet states, upon excitation with UV irradiation, followed by inter conversion (IC) and intersystem crossing (ISC) to its low-energy excited triplet state, and, then, subsequent energy transfer to the Eu^3+^ and Tb^3+^ excited states that relax to the ground levels emitting the characteristic 4*f* emission [64]. As it is well known, the energy differences between the ligands singlet and triplet excited states and between the ligand triplet excited state and the Ln^3+^ excited resonance levels are vital factors that affect the energy transfer efficiency. According to the Reinhoudt’s empirical rule [65], in order to get an efficient energy transfer the energy difference between the ligands singlet and triplet excited state should be larger than 5000 cm^−1^, whereas the energy differences between the ligand triplet excited state and the Ln^3+^ excited resonance levels should be 2500–4000 cm^−1^ for Eu^3+^ and 2500–4500 cm^−1^ for Tb^3+^, Latva’s empirical rule [66].

The low-energy singlet and triplet excited states of Oba were estimated from the absorption edge in the UV–vis spectrum and the lowest emission peak in the low temperature phosphorescence spectrum of the Gd^3+^-based complex to be around 33,300 cm^−1^ (300 nm) and 26,250 cm^−1^ (381 nm), respectively [43]. Therefore, the energy difference between the singlet and triplet excited state of Oba is more than 5000 cm^−1^, anticipating an effective intersystem crossing. Although the energy differences between the triplet excited state of Oba and the resonance Tb^3+^ (^5^D_4_, 20,500 cm^−1^) and Eu^3+^ (^5^D_0_, 17,500 cm^−1^) levels are 5750 and 8750 cm^−1^, respectively, Oba can still sensitize Ln^3+^ through the bridge of Phen since the triplet energy level of Phen locates at around 22,100 cm^−1^ [67].

**Scheme 2:**
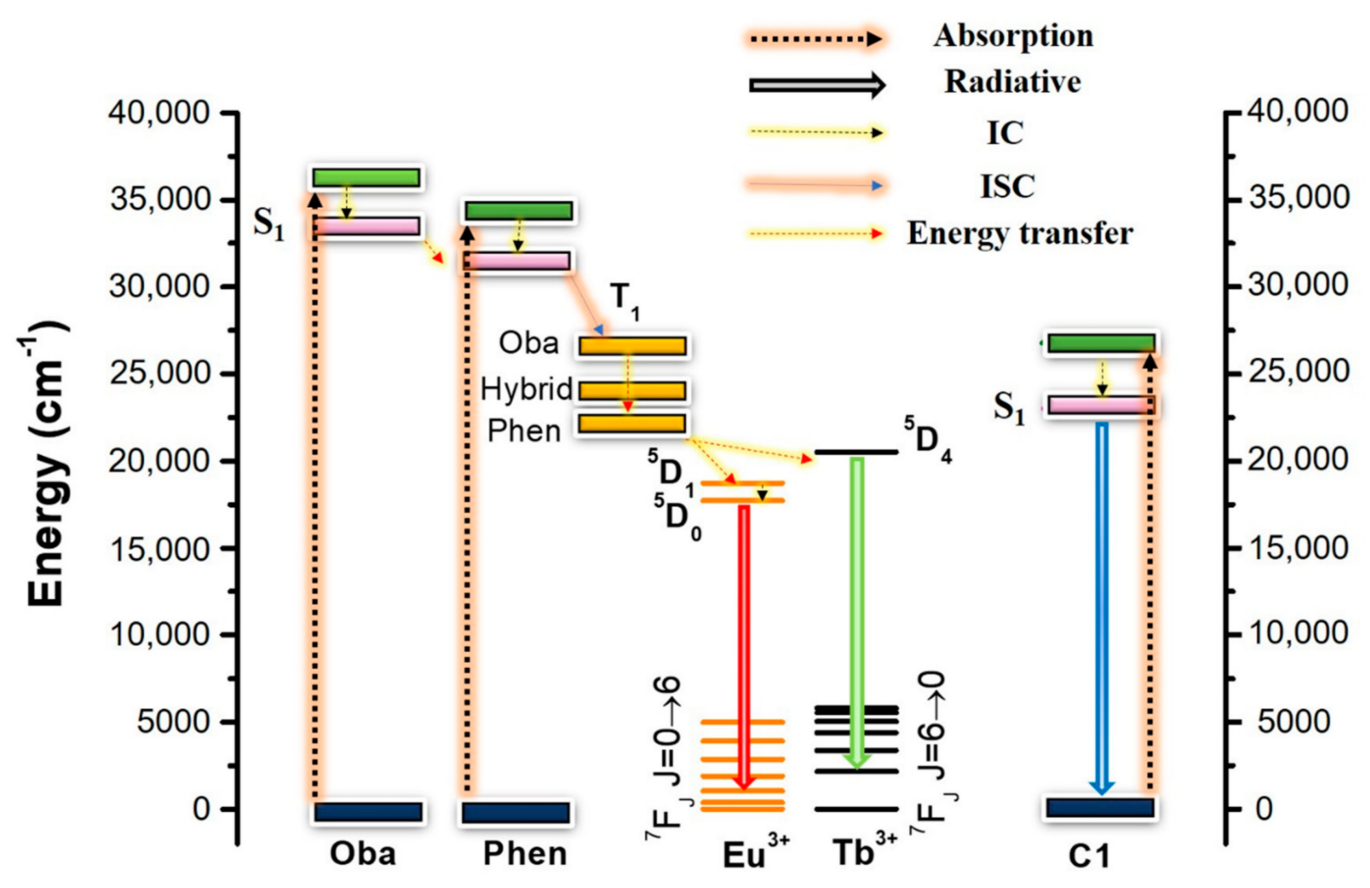
Part of the energy levels of Tb^3+^ and Eu^3+^ ions, Phen and Oba organic ligands, d-U(600) matrix and C1 dye. The absorption pathways, IC and ISC processes, energy transfer, and radiative emission are also depicted.

To check whether there is Tb^3+^-to-Eu^3+^ energy transfer, the luminescent decay curves were measured for Tb^3+^-doped and Eu^3+^/Tb^3+^-codoped d-U(600) (Figure S14 in Supplementary Material). The energy transfer efficiency was estimated from the ^5^D_4_ lifetimes measured with and without Eu^3+^ cooping. The very low energy transfer efficiency obtained (2.6%) indicates that in this case there is almost no energy transfer between the two metal ions, as the analysis of the Eu^3+^ excitation spectrum (Figure S7b) already pointed out. Based on the above discussion, and in accord with the energy of the low-energy excited level of the d-U(600) matrix (at around 24,000 cm^−1^ [68]), the following energy transfer paths for Ln^3+^, sensitization can occur: (i) T_1_ of Oba to T_1_ of Phen followed by T_1_ of Phen to Ln^3+^, and (ii) T_1_ of Oba to d-U(600), followed by the di-ureasil matrix-to-T_1_ of Phen and then to Ln^3+^ and di-ureasil matrix-to-Ln^3+^, as shown in Scheme 2. Accordingly, and upon excitation under UV light, white-light emission is achieved combining the blue color emission from C1 with the green and red ones from Tb^3+^ and Eu^3+^, respectively.

## 4. Conclusions

In this paper, whit-light emitting d-U(600) di-ureasils were prepared by in situ sol–gel technique through hydrolysis and condensation of the precursor in the presence of Ln^3+^ (Ln = Eu, Tb) ions, Oba and Phen organic ligands and C1 dye. The Phen organic ligand can directly transfer energy to the Ln^3+^ ions, while Oba can also sensitize the Ln^3+^ emissions via Oba–host–Ln^3+^ and/or Oba–Phen–Ln^3+^ energy transfer paths. Furthermore, there is nearly no Tb^3+^-to-Eu^3+^ and C1-to-Ln^3+^ energy transfer. Consequently, the resulting luminescent di-ureasils exhibit distinct blue, green, and red colors and, thus, white-light emission has been obtained at 305 nm irradiation with absolute quantum yield of 0.202 ± 0.020. The fine-tuned emissions of the blue, green, and red of the dopants opens the possibility to synthesize other hybrids with efficient warm-white light emitting features. The in situ sol–gel technique demonstrates the excellent effect at dispersing Ln^3+^ complexes with polycarboxylate organics as ligands which can easily form insoluble state due to the networking character. Furthermore, the in situ sol–gel approach, with several unique features, such as mild reaction conditions, high product homogeneity and easy processing and shaping, is a promising strategy for preparing Ln^3+^/dye-doped white-light emitting materials that are not dissoluble and/or decomposed during conventional sol–gel method. 

## Supplementary Materials

The following are available online at http://www.mdpi.com/1996-1944/11/11/2246/s1, Materials and Methods, Scheme S1: Schematic representation of synthesis process of doped di-ureasil. Figure S1. XRD patterns of (1) d-U(600), (2) Eu-I, (3) Tb-I, (4) EuTb-I, (5) EuTbC1-I, (6) EuTbC1-II, and (7) EuTbC1-III; Figure S2: FT-IR spectra of (1) d-U(600), (2) Eu-I, (3) Tb-I, (4) EuTb-I, (5) EuTbC1-I, (6) EuTbC1-II, and (7) EuTbC1-III; Figure S3: TG curves of d-U(600), Eu-I, Tb-I, EuTb-I, EuTbC1-I, EuTbC1-II, and EuTbC1-III; Figure S4: Calculated UV–vis absorption spectrum for C1 by TD-DFT method at B3LYP/6-31G(d) level of theory with polarized continuum model in EtOH; Figure S5: Contour plots of the highest occupied molecular orbital (HOMO, left) and the lowest unoccupied molecular orbital (LUMO, right) of C1; Figure S6: (a) Excitation spectra of (1) Eu-I and (2) Tb-I monitored at 615 and 545 nm, respectively; (b) emission spectra of Eu-I excited at (1) 275, (2) 295, (3) 310, and (4) 350 nm; (c) emission spectra of Tb-I excited at (1) 275, (2) 295, and (3) 350 nm; Figure S7: (a) Emission spectra of EuTb-I excited at 295, 350, 365, 370, and 380 nm; (b) excitation spectra of EuTb-I monitored at (1) 460, (2) 544, and (3) 615 nm; Figure S8: (a) Emission spectra of EuTbC1-I excited at different wavelength from 295 to 365 nm; (b) excitation spectra of EuTbC1-I monitored at (1) 430, (2) 545, and (3) 615 nm; Figure S9: (a) Emission spectra of EuTbC1-II excited at different wavelength from 275 to 365 nm; (b) excitation spectra of EuTbC1-II monitored at (1) 430, (2) 545, and (3) 615 nm; Figure S10. (a) Emission spectra of EuTbC1-III excited at different wavelength from 295 to 350 nm; (b) excitation spectra of EuTbC1-III monitored at (1) 440, (2) 545, and (3) 615 nm; Figure S11: CIE Chromaticity color diagram showing the EuTbC1-I emission color coordinates (300 K) at different irradiation times; Figure S12: Excitation spectra (270–400 nm) of EuTb-I, EuTbC1-I, and EuTbC1-II all monitored at (a) 545 nm and (b) 615 nm; Figure S13: UV–vis absorption (left) and emission spectra (right, *λ*_em_ = 373 nm) spectra of C1 in EtOH (1.0 × 10^−5^ M); Figure S14: Emission decay curves of (a) Tb-I and (b) EuTb-I, both excited at 295 nm and monitored at 545 nm; Table S1: Components for synthesis of di-ureasils d-U(600)s doped with Ln(NO_3_)_3_, Oba, Phen and dye C1; Table S2: Absolute quantum yields for some Ln based white-light emission materials.

## References

[1] Cho J., Park J.H., Kim J.K., Schubert E.F. (2017). White light-emitting diodes: History, progress, and future. Laser Photonics Rev..

[2] Schubert E.F., Kim J.K. (2005). Solid-state light sources getting smart. Science.

[3] Carlos L.D., Ferreira R.A.S., De Zea Bermudez V., Ribeiro S.J.L. (2009). Lanthanide-containing light-emitting organic–inorganic hybrids: A bet on the future. Adv. Mater..

[4] Bünzli J.C.G., Piguet C. (2005). Taking advantage of luminescent lanthanide ions. Chem. Soc. Rev..

[5] Escribano P., Julián-López B., Planelles-Aragó J., Cordoncillo E., Viana B., Sanchez C. (2008). Photonic and nanobiophotonic properties of luminescent lanthanide-doped hybrid organic–inorganic materials. J. Mater. Chem..

[6] Sutar P., Suresh V.M., Maji T.K. (2015). Tunable emission in lanthanide coordination polymer gels based on a rationally designed blue emissive gelator. Chem. Commun..

[7] Laishram R., Bhowmik S., Maitra U. (2015). White light emitting soft materials from off-the-shelf ingredients. J. Mater. Chem. C.

[8] Yang J., Yan Y., Hui Y., Huang J. (2017). White emission thin films based on rationally designed supramolecular coordination polymers. J. Mater. Chem. C.

[9] Chen W., Fan R., Zhang H., Dong Y., Wang P., Yang Y. (2017). Tunable white-light emission PMMA-supported film materials containing lanthanide coordination polymers: Preparation, characterization, and properties. Dalton Trans..

[10] Narayana Y.S.L.V., Basak S., Baumgarten M., Müllen K., Chandrasekar R. (2013). White-emitting conjugated polymer/inorganic hybrid spheres: Phenylethynyl and 2,6-bis(pyrazolyl)pyridine copolymer coordinated to Eu(tta)_3_. Adv. Funct. Mater..

[11] Balamurugan A., Reddy M.L.P., Jayakannan M. (2009). Single polymer photosensitizer for Tb^3+^ and Eu^3+^ ions: An approach for white light emission based on carboxylic-functionalized poly(*m*-phenylenevinylene)s. J. Phys. Chem. B.

[12] Lian X., Yan B. (2016). Novel core–shell structure microspheres based on lanthanide complexes for white-light emission and fluorescence sensing. Dalton Trans..

[13] Wu J., Zhang H., Du S. (2016). Tunable luminescence and white light emission of mixed lanthanide–organic frameworks based on polycarboxylate ligands. J. Mater. Chem. C.

[14] Hasegawa Y., Nakanishi T. (2015). Luminescent lanthanide coordination polymers for photonic applications. RSC Adv..

[15] Cui Y., Yue Y., Qian G., Chen B. (2011). Luminescent functional metal–organic frameworks. Chem. Rev..

[16] Jung H.S., Kim Y.J., Ha S.W., Lee J.K. (2013). White light-emitting diodes using thermally and photochemically stable fluorescent silica nanoparticles as color-converters. J. Mater. Chem. C.

[17] SeethaLekshmi S., Ramya A.R., Reddy M.L.P., Varughese S. (2017). Lanthanide complex-derived white-light emitting solids: A survey on design strategies. J. Photochem. Photobiol. C.

[18] Huo R., Li X., Ma D. (2015). Lanthanide coordination frameworks constructed from 1,3-benzenedicarboxylate, oxalate and 1,10-phenanthroline: Crystal structure, multicolor luminescence and high-efficiency white light emission. CrystEngComm.

[19] Zhang H., Shan X., Zhou L., Lin P., Li R., Ma E., Guo X., Du S. (2013). Full-colour fluorescent materials based on mixed-lanthanide(iii) metal–organic complexes with high-efficiency white light emission. J. Mater. Chem. C.

[20] Ramya A.R., Varughese S., Reddy M.L.P. (2014). Tunable white-light emission from a mixed lanthanide (Eu^3+^ Gd^3+^ Tb^3+^) coordination polymers derived from 4-(dipyridin-2-yl)aminobenzoate. Dalton Trans..

[21] Dang S., Zhang J.H., Sun Z.M. (2012). Tunable emission based on lanthanide(iii) metal–organic frameworks: An alternative approach to white light. J. Mater. Chem..

[22] Song S., Li X., Zhang Y.H., Huo R., Ma D. (2014). White light emission by a lanthanide doped Sm(III) framework constructed from 4-sulfobenzoate and 1H-imidazo[4,5-f][1,10]-phenanthroline. Dalton Trans..

[23] Ma M.L., Ji C., Zang S.Q. (2013). Syntheses, structures, tunable emission and white light emitting Eu^3+^ and Tb^3+^ doped lanthanide metal–organic framework materials. Dalton Trans..

[24] Ma X., Li X., Cha Y.E., Jin L.P. (2012). Highly thermostable one-dimensional lanthanide(III) coordination polymers constructed from benzimidazole-5,6-dicarboxylic acid and 1,10-phenanthroline: Synthesis, structure, and tunable white-light emission. Cryst. Growth Des..

[25] Zhang F., Yan P., Li H., Zou X., Hou G., Li G. (2014). Towards full-color-tunable emission of two component Eu(III)-doped Gd(III) coordination frameworks by the variation of excitation light. Dalton Trans..

[26] Rao X., Huang Q., Yang X., Cui Y., Yang Y., Wu C., Chen B., Qian G. (2012). Color tunable and white light emitting Tb^3+^ and Eu^3+^ doped lanthanide metal–organic framework materials. J. Mater. Chem..

[27] Liu J., Sun W., Liu Z. (2016). White-light emitting materials with tunable luminescence based on steady Eu(III) doping of Tb(III) metal–organic frameworks. RSC Adv..

[28] Liu Z.F., Wu M.F., Wang S.H., Zheng F.K., Wang G.E., Chen J., Xiao Y., Wu A.Q., Guo G.C., Huang J.S. (2013). Eu^3+^-Doped Tb^3+^ metal–organic frameworks emitting tunable three primary colors towards white light. J. Mater. Chem. C.

[29] Song S., Li X., Zhang Y.-H. (2013). White light emission of an Eu(III)-doped Gd(III) complex with 3-sulfobenzoate and 1H-imidazo[4,5-f][1,10]-phenanthroline. Dalton Trans..

[30] He G., Guo D., He C., Zhang X., Zhao X., Duan C. (2009). A color-tunable europium complex emitting three primary colors and white light. Angew. Chem. Int. Ed..

[31] Shelton A.H., Sazanovich I.V., Weinstein J.A., Ward M.D. (2012). Controllable three-component luminescence from a 1,8-naphthalimide/Eu(III) complex: White light emission from a single molecule. Chem. Commun..

[32] Yang Q.Y., Wu K., Jiang J.J., Hsu C.W., Pan M., Lehn J.M., Su C.Y. (2014). Pure white-light and yellow-to-blue emission tuning in single crystals of Dy(III) metal–organic frameworks. Chem. Commun..

[33] Wang J.H., Chorazy S., Nakabayashi K., Sieklucka B., Ohkoshi S. (2018). Achieving white light emission and increased magnetic anisotropy by transition metal substitution in functional materials based on dinuclear Dy^III^(4-pyridone)[M^III^(CN)_6_]^3−^ (M=Co, Rh) molecules. J. Mater. Chem. C.

[34] Zhang X.P., Wang D.G., Su Y., Tian H.R., Lin J.J., Feng Y.L., Cheng J.W. (2014). Luminescent 2D bismuth–cadmium–organic frameworks with tunable and white light emission by doping different lantahnide ions. Dalton Trans..

[35] Dar W.A., Ahmed Z., Iftikhar K. (2018). Cool white light emission from the yellow and blue emission bands of the Dy(III) complex under UV-excitation. J. Photochem. Photobiol. A.

[36] Kerbellec N., Kustaryono D., Haquin V., Etienne M., Daiguebonne C., Guillou O. (2009). An unprecedented family of lanthanide-containing coordination polymers with highly tunable emission properties. Inorg. Chem..

[37] Song T., Zhang G., Cui Y., Yang Y., Qian G. (2016). Encapsulation of coumarin dye within lanthanide MOFs as highly efficient white-light-emitting phosphors for white LEDs. CrystEngComm.

[38] Wen Y., Sheng T., Zhu X., Zhuo C., Su S., Li H., Hu S., Zhu Q.L., Wu X. (2017). Introduction of red-green-blue fluorescent dyes into a metal–organic framework for tunable white light emission. Adv. Mater..

[39] Wang Z., Wang Z., Lin B., Hu X., Wei Y., Zhang C., An B., Wang C., Lin W. (2017). Warm-white-light-emitting diode based on a dye-loaded metal-organic framework for fast white-light communication. ACS Appl. Mater. Interfaces.

[40] De Zea Bermudez V., Carlos L.D., Alcácer L. (1999). Sol–gel derived urea cross-linked organically modified silicates. 1. room temperature mid-infrared spectra. Chem. Mater..

[41] Carlos L.D., De Zea Bermudez V., Ferreira R.A.S., Marques L., Assunção M. (1999). Sol–gel derived urea cross-linked organically modified silicates. 2. blue-light emission. Chem. Mater..

[42] Ma D., Li X., Huo R. (2014). A high-efficiency white light-emitting lanthanide–organic framework assembled from 4,4′-oxybis(benzoic acid), 1,10-phenanthroline and oxalate. J. Mater. Chem. C.

[43] Fang M., Fu L.S., Correia S.F.H., Ferreira R.A.S., Carlos L.D. (2018). Highly efficient luminescent polycarboxylate lanthanide complexes incorporated into di-ureasils by an in-situ sol–gel Process. Polymers.

[44] Diraison M., Millie P., Pommeret S., Gustavsson T., Mialocq J.C. (1998). Solvation structure of coumarin 1 in acetonitrile: Role of the electrostaticsolute-solvent potential. Chem. Phys. Lett..

[45] Gupta M., Maity D.K., Singh M.K., Nayak S.K., Ray A.K. (2012). Supramolecular interaction of coumarin 1 dye with cucurbit[7]uril as host: Combined experimental and theoretical study. J. Phys. Chem. B.

[46] Gangopadhyay S., Pleil M.W., Borst W.L. (1987). Study of energy transfer in a solution of coumarin 460 and rhodamine 6G by time-resolved laser-induced fluorescence spectroscopy. J. Lumin..

[47] McCamy C.S. (1992). Correlated color temperature as an explicit function of chromaticity coordinates. Color Res. Appl..

[48] Fu L.S., Ferreira R.A.S., Fernandes M., Nunes S.C., De Zea Bermudez V., Hungerford G., Rocha J., Carlos L.D. (2008). Photoluminescence and quantum yields of organic/inorganic hybrids prepared through formic acid solvolysis. Opt. Mater..

[49] Borie B. (1965). X-ray diffraction in crystals, imperfect crystals, and amorphous bodies. J. Am. Chem. Soc..

[50] Skrovanek D.J., Howe S.E., Painter P.C., Coleman M.M. (1985). Hydrogen bonding in polymers: Infrared temperature studies of an amorphous polyamide. Macromolecules.

[51] Coleman M.M., Lee K.H., Skrovanek D.J., Painter P.C. (1986). Hydrogen bonding in polymers. 4. infrared temperature studies of a simple polyurethane. Macromolecules.

[52] Fu L.S., Ferreira R.A.S., Silva N.J.O., Carlos L.D., De Zea Bermudez V., Rocha J. (2004). Photoluminescence and quantum yields of urea and urethane cross-linked nanohybrids derived from carboxylic acid solvolysis. Chem. Mater..

[53] Dingemans G., van Helvoirt C.A.A., van de Sanden M.C.M., Kessels W.M.M. (2011). Plasma-assisted atomic layer deposition of low temperature SiO_2_. ECS Trans..

[54] Lima P.P., Almeida Paz F.A., Ferreira R.A.S., De Zea Bermudez V., Carlos L.D. (2009). Ligand-assisted rational design and supramolecular tectonics toward highly luminescent Eu^3+^ containing organic–inorganic hybrids. Chem. Mater..

[55] Fernandes M., De Zea Bermudez V., Ferreira R.A.S., Carlos L.D., Martins N.V. (2008). Incorporation of the Eu(tta)_3_(H_2_O)_2_ complex into a co-condensed d-U(600)/d-U(900) matrix. J. Lumin..

[56] Accorsi G., Listorti A., Yoosaf K., Armaroli N. (2009). 1,10-Phenanthrolines: Versatile building blocks for luminescent molecules, materials and metal complexes. Chem. Soc. Rev..

[57] Kitazawa N., Miyagawa S., Date K., Aroonjaeng W., Aono M., Watanabe Y. (2009). Optical properties of dye-doped deoxyribonucleic acid films. J. Mater. Sci..

[58] Irfanullah M., Iftikhar K. (2011). The correlation between f-f absorption and sensitized visible light emission of luminescent Pr(III) complexes: Role of solvents and ancillary ligands on sensitivity. J. Lumin..

[59] Lima P.P., Ferreira R.A.S., Júnior S.A., Malta O.L., Carlos L.D. (2009). Terbium(III)-containing organic–inorganic hybrids synthesized through hydrochloric acid catalysis. J. Photochem. Photobiol. A Chem..

[60] Correia S.F.H., Lima P.P., Pecoraro E., Ribeiro S.J.L., André P.S., Ferreira R.A.S., Carlos L.D. (2016). Scale up the collection area of luminescent solar concentrators towards metre-length flexible waveguiding photovoltaics. Prog. Photovolt. Res. Appl..

[61] Nolasco M.M., Vaz P.M., Freitas V.T., Lima P.P., André P.S., Ferreira R.A.S., Vaz P.D., Ribeiro-Claro P., Carlos L.D. (2013). Engineering highly efficient Eu(III)-based tri-ureasil hybrids toward luminescent solar concentrators. J. Mater. Chem. A.

[62] Ferreira R.A.S., Carlos L.D., De Zea Bermudez V. (1999). Excitation energy dependence of luminescent sol–gel organically modified silicates. Thin Solid Films.

[63] Carlos L.D., Ferreira R.A.S., Pereira R.N., Assunção M., De Zea Bermudez V. (2004). White-light emission of amine-functionalized organic/inorganic hybrids: Emitting centers and recombination mechanisms. J. Phys. Chem. B.

[64] Carnall W.T., Goodman G.L., Rajnak K., Rana R.S. (1989). A systematic analysis of the spectra of the lanthanides doped into single crystal LaF_3_. J. Chem. Phys..

[65] Steemers F.J., Verboom W., Reinhoudt D.N., van der Tol E.B., Verhoeven J.W. (1995). New sensitizer-modified calix[4]arenes enabling near-UV excitation of complexed luminescent lanthanide ions. J. Am. Chem. Soc..

[66] Latva M., Takalo H., Mukkala V.M., Matachescu C., Rodríguez-Ubis J.C., Kankare J. (1997). Correlation between the lowest triplet state energy level of the ligand and lanthanide(III) luminescence quantum yield. J. Lumin..

[67] Bala M., Kumar S., Taxak V.B., Boora P., Khatkar S.P. (2016). Optical features of efficient europium(III) complexes with β-diketonato and auxiliary ligands and mechanistic investigation of energy transfer process. J. Fluoresc..

[68] Lima P.P., Ferreira R.A.S., Freire R.O., Almeida Paz F.A., Fu L.S., Alves S., Carlos L.D., Malta O.L. (2006). Spectroscopic study of a UV-photostable organic–inorganic-hybrids incorporating an Eu^3+^ β-diketonate complex. Chem. Phys. Chem..

