# Supplementary material for: White-Light Emitting Di-Ureasil Hybrids
Source: Materials (Basel). 2018 Nov 12;11(11):2246. doi: 10.3390/ma11112246 (PMC6266990; doi:10.3390/ma11112246)
Supplement: Supplementary file 1 [file materials-11-02246-s001.pdf]

materials

Supplementary Material

White-Light Emitting Di-Ureasil Hybrids

Ming Fang, Lianshe Fu, Rute A. S. Ferreira and Luís D. Carlos

Department of Physics, CICECO-Aveiro Institute of Materials, University of Aveiro, 3810-193 Aveiro, Portugal

Materials and Methods

Materials

The diamine α,ω-diaminepoly(oxyethylene-co-oxypropylene) (ED-600, Huntsman, Barcelona, Spain), 3-isocyanateproplytriethoxysilane (ICPTES, 95%, Aldrich, Algés, Portugal), Oba (98%, ABCR), Phen (99%, Alfa Aesar, Karlsruhe, Germany) and coumarin 1 (C1, 98%, Alfa Aesar, Karlsruhe, Germany) are all commercially available. $Eu(NO_3)_3$ and $Tb(NO_3)_3$ were obtained by dissolving of Eu_2O_3 and Tb_4O_7 (Shanghai Yuelong New Material Co., Ltd., China) in HNO_3, respectively. Tetrahydrofuran (THF 99%, Sigma–Aldrich, Algés, Portugal), absolute ethanol (EtOH, Sigma–Aldrich, Algés, Portugal) and dimethylformamide (DMF, 99.99%, Fisher Chemical, Oeiras Portugal) were used as solvents and hydrochloride (HCl, Sigma–Aldrich, Algés, Portugal) was used as catalyst in sol–gel synthesis. All chemicals were used as received without purifications.

Synthesis

The non-hydrolyzed di-ureasil d-UPTES(600) was synthesized at room temperature according to the literature [S1] using THF as solvent, followed by evaporation of THF under vacuum. The undoped di-ureasil, d-U(600), was prepared by hydrolysis and polycondensation of d-UPTES(600) using EtOH as the cosolvent and HCl as the catalyst for sol–gel process at room temperature. The resulting gel was dried at 50 °C.

Generally, the incorporation of the optically active centers was performed as follows. One gram of d-UPTES(600) precursor was mixed with 0.96 mL of EtOH and stirred at room temperature. Then a mixture containing $Ln(NO_3)_3$ (Ln = Eu or Tb) in EtOH, Oba in DMF (ultrasonic treatment) and Phen or 3.5×10^{-3} mmol of C1 in EtOH was added. The molar ratio of d-UPTES(600):Ln is 1:0.06 and Ln:Oba:Phen is 1:1:1. Finally, 98.7 μL of HCl-acidified water (pH = 2) was added to catalyze the sol–gel reaction. The molar ratio of d-UPTES(600):H_2O is 1:6. The resulting d-U(600) singly doped with Eu^{3+}, Tb^{3+} or C1 were dried at 50 °C, and denoted as Eu-I, Tb-I, and C1-I, respectively.

Synthesis of di-ureasil d-U(600) co-doped with Ln^{3+} (Eu^{3+} and Tb^{3+}) complex is similar to that of Eu-I, Tb-I, and C1-I except that Eu^{3+}-and Tb^{3+}-based complexes were incorporated into d-UPTES(600) simultaneously. The molar ratio of Eu:Tb is 3:7. The resultant di-ureasil was designated as EuTb-I.

Synthesis of di-ureasil d-U(600)s triply doped with Ln^{3+} (Eu^{3+} and Tb^{3+}) complex and C1. In a typical way, 1.0 g of d-UPTES(600) (0.914 mmol) was mixed with 0.96 mL of EtOH. $Ln(NO_3)_3$ (Ln^{3+} = Eu^{3+} and Tb^{3+}), Oba and Phen in different solvents were added with the same molar ratio of di-ureasil d-U(600) doubly doped with Ln^{3+} (Eu and Tb) complex. Then three different amounts of C1 (8.7×10^{-5}, 8.7×10^{-4} and 3.5×10^{-3} mmol) in EtOH were added to the above mixture. A diluted HCl (pH = 2) was added to speed up the sol–gel reaction. The resulting di-ureasils d-U(600)s containing

Ln^{3+} (Eu^{3+} and Tb^{3+}) and different C1 contents are named as EuTbC1-I, EuTbC1-II and EuTbC1-III, respectively. After hydrolysis and polycondensation reactions of the precursor in the presence of $Ln(NO_3)_3$ (Eu^{3+} and Tb^{3+}), Oba, Phen and C1 using HCl as the catalyst, the di-ureasil monoliths containing in-situ Ln complex and dye were obtained, as shown in Scheme S2. At the doping concentration (6.0 mol% relative to the di-ureasil) in this work, the resulting di-ureasils display excellent transparency and mechanical flexibility for ease of processing. Furthermore, these hybrid materials show high and nearly no absorption in the UV and visible regions, respectively, which will be discussed in the following part, making them promising materials for optoelectronic applications.

Schemes

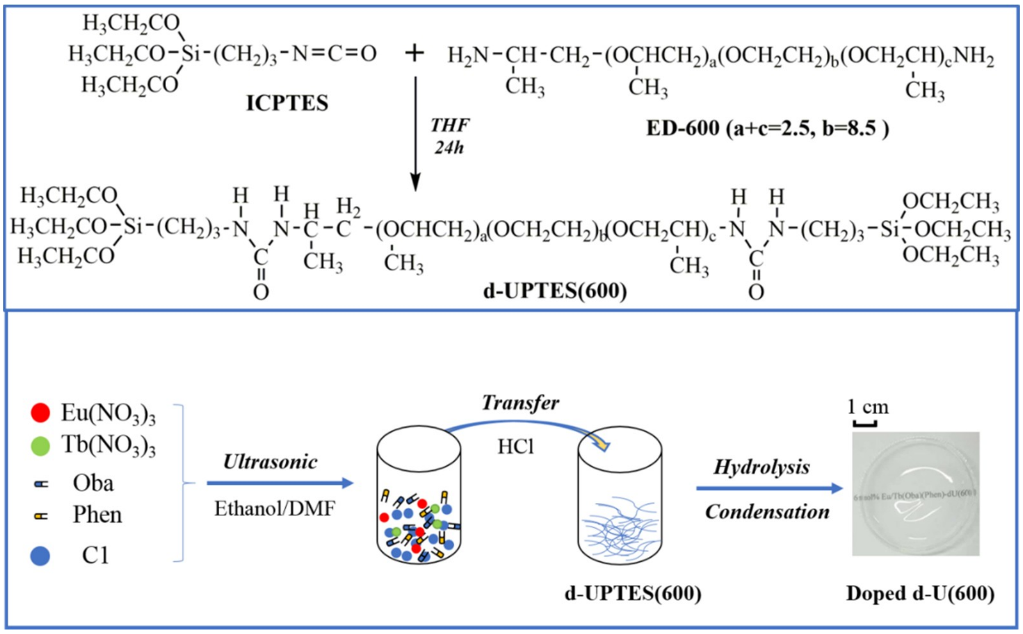

Scheme S1. Schematic representation of synthesis process of doped di-ureasil.

Tables

Table S1. Components for synthesis of di-ureasils d-U(600)s doped with $Ln(NO_3)_3$, Oba, Phen and dye C1.

Samples	Eu-I	Tb-I	EuTb-I	EuTbC1-I	EuTbC1-II	EuTbC1-III	C1-I
0.1 M $Eu(NO_3)_3$ μL (mmol)	548.4 (0.0548)		164.5 (0.0165)	164.5 (0.0165)	164.5 (0.0165)	164.5 (0.0165)	
0.1 M $Tb(NO_3)_3$ μL (mmol)		548.4 (0.0548)	383.9 (0.0384)	383.9 (0.0384)	383.9 (0.0384)	383.9 (0.0384)	
Oba mg (mmol)	14.2 (0.0550)	14.2 (0.0550)	14.2 (0.0550)	14.2 (0.0550)	14.2 (0.0550)	14.2 (0.0550)	
Phen mg (mmol)	9.9 (0.0550)	9.9 (0.0550)	9.9 (0.0550)	9.9 (0.0550)	9.9 (0.0550)	9.9 (0.0550)	
0.00432 M C1 μL (mmol)				20 (8.7×10^{-5})	200 (8.7×10^{-4})	800 (3.5×10^{-3})	800 (3.5×10^{-3})
DMF mL	0.75	0.75	0.75	0.75	0.75	0.75	

Note: For syntheses of d-U(600) and other di-ureasils codoped with Ln, Oba, Phen and dye C1, 1.0 g of d-UPTES(600) precursor, 0.96 mL of EtOH and 98.7 μL of HCl (pH=2) were added.

Table S2. Absolute quantum yields for some Ln^{3+} based white-light emission materials.

System	Materials	Emission colour coordinates (x,y)	QY	Reference
MOFs	ZJU-1:(1.5%)Tb^{3+},(2.0%)Eu^{3+}	(0.31,0.33)	0.068	S2
	(10%)Eu-SMOF-1	(0.37,0.30)	0.043	S3
	(0.183)Eu^{3+}/(0.408)Tb^{3+}@Zn(II)-MOF	(0.34,0.33)	0.08	S4
	(1:2)Eu^{3+}/Tb^{3+}@porous MOF	(0.36,0.32)	0.113	S5
	[(3.5 wt%)Ir$(ppy)_2$(bpy)]$^+$@ porous MOF	(0.31,0.33)	0.204	S5
	NENU-524-⊃[(3.86wt%) Ir$(ppy)_2$(bpy)]$^+$	(0.30,0.34)	0.152	S6
	$(Yb_{0.73}Tb_{0.25}Eu_{0.02})_2(1,3\text{-}BDC)_2(phen)_2(ox)(H_2O)$	(0.35,0.36)	0.20	S7
	$[Sm_{99.83}Eu_{0.17}$(4-SBA)-(IP)OH]·1.5H_2O	(0.34,0.32)	0.034	S8
	(5%)Eu^{3+}-{$[TbOH(H_2O)_6][Zn_2Tb_4$(4-Htbca)$_2$-(4-t bca)$_8(H_2O)_{12}]$}N·6nH_2O	(0.33,0.33)	0.114	S9
	$[NaGd_{0.90}Eu_{0.10}(C_8N_2O_8(H_2O)_3)]\cdot H_2O$ or $[NaLn(pztc)(H_2O)_3]\cdot H_2O$	(0.39,0.38)	0.076	S10
	$[Dy(TETP)(NO_3)_3]\cdot 4H_2O$	(0.33,0.35)	0.07	S11
MOFs/Dye	$Eu_{0.05}Tb_{0.95}$BPT⊃C460	(0.32,0.34)	0.4332	S12
	ZJU 28⊃(0.02wt%)DSM/(0.06%)AF	(0.34,0.32)	0.174	S13
	HSB-W1⊃DCM/C6a/CBS-127	(0.31,0.32)	0.26	S14
Polymers	(PEI/TPE/Tb^{3+})15/(PEI/Ln^{3+}–L_2EO_4)$_5$	(0.34,0.35)	0.1174	S15
	$W_{(Eu,Gd,Tb)}$-PMMA	(0.33,0.35)	0.176	S16
Gels	LMWG:Tb^{III}:Eu^{III}	(0.28,0.34)	0.05	S17
	Tb/Eu/Zn-DHN@cholate gel	(0.33,0.34)	0.07	S18
Complexes	$[Gd_{99.66}Eu_{0.34}$(3-SBA)(IP)OH$(H_2O)]\cdot H_2O$	(0.34,0.33)	0.0992	S19
	$[H_2NMe_2]_3[Gd_{0.9365}Eu_{0.370}Tb_{0.0265}(L)_3]$	(0.33,0.34)	0.62	S20

Figures

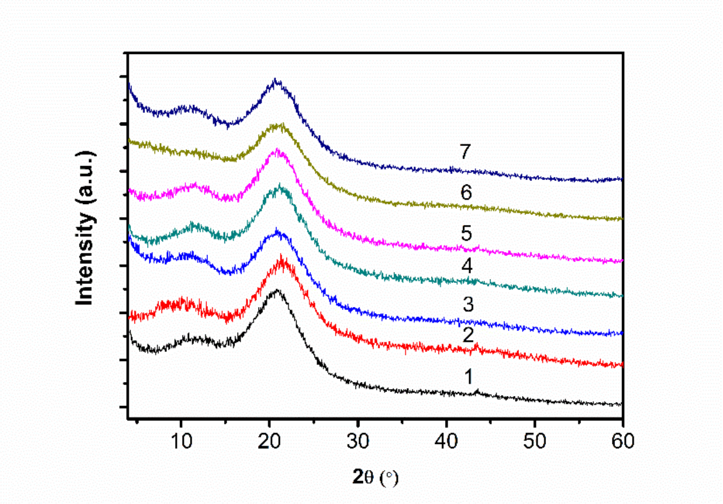

Figure S1. XRD patterns of (1) d-U(600), 2) Eu-I, 3) Tb-I, 4) EuTb-I, 5) EuTbC1-I, 6) EuTbC1-II and 7) EuTbC1-III.

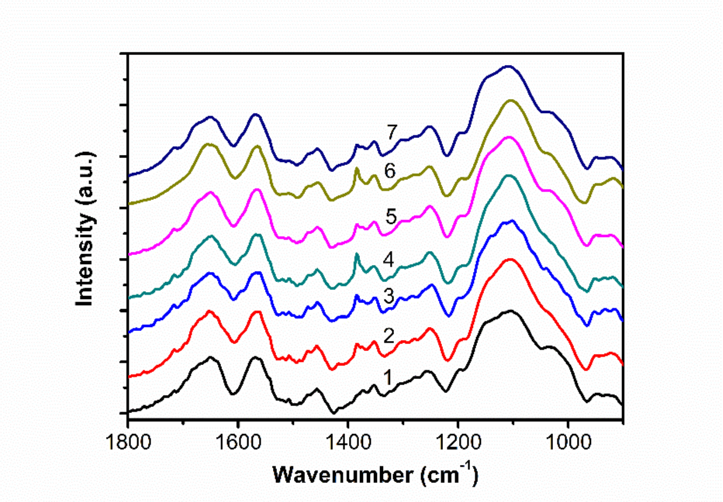

Figure S2. FT-IR spectra of 1) d-U(600), 2) Eu-I, 3) Tb-I, 4) EuTb-I, 5) EuTbC1-I, 6) EuTbC1-II and 7) EuTbC1-III.

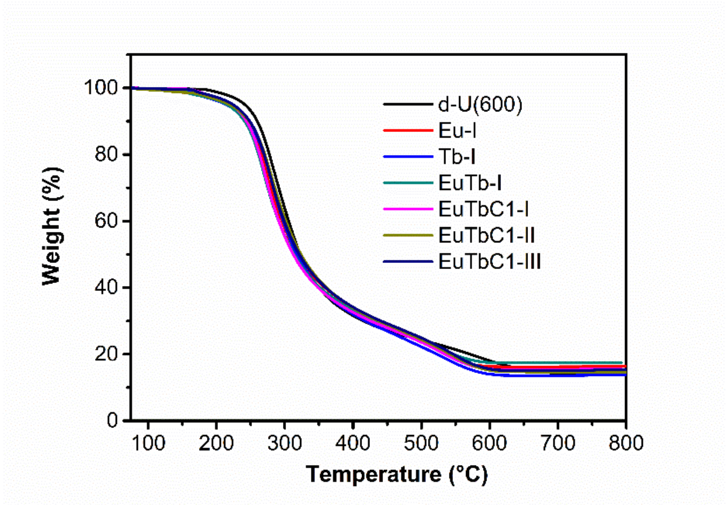

Figure S3. TG curves of d-U(600), Eu-I, Tb-I, EuTb-I, EuTbC1-I, EuTbC1-II and EuTbC1-III.

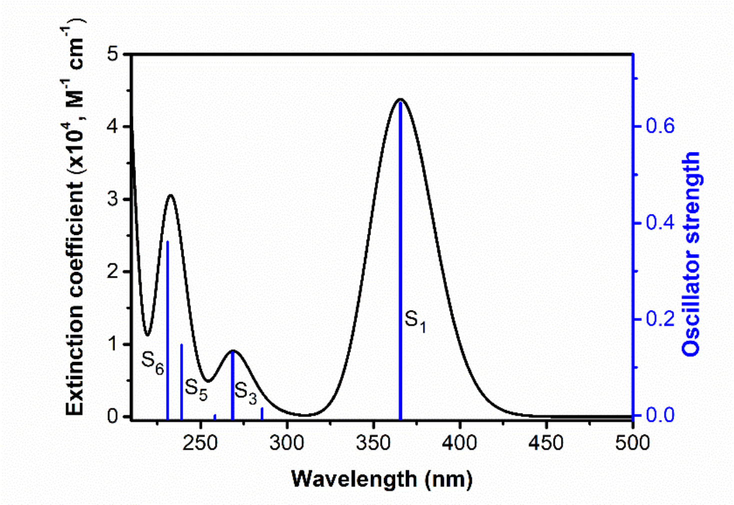

Figure S4. Calculated UV–Vis absorption spectrum for C1 by TD-DFT method at B3LYP/6-31G(d) level of theory with polarized continuum model in EtOH using Gaussian 09 package [S21]. The vertical lines indicate the oscillator strength of each excited state. The calculated first excited state S_1 at 365 nm is in agreement well with the experimental maximum absorption wavelength (373 nm) in EtOH. Since $S_0 \rightarrow S_1$ transition is predominantly involved the electron promotion from HOMO to LUMO (98.9%), according to the contour plots of HOMO and LUMO (Figure S5), this transition can be ascribed to $\pi \rightarrow \pi^*$ transition with charge transfer characteristics.

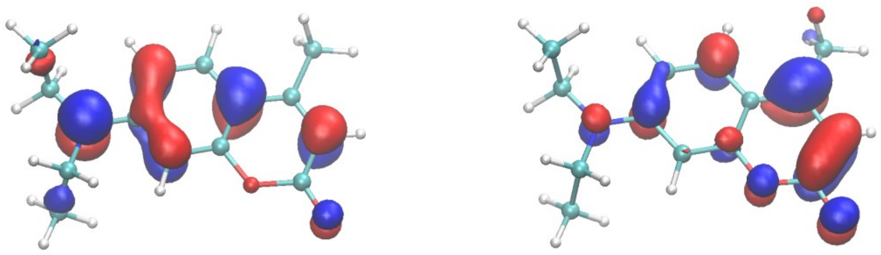

Figure S5. Contour plots of the highest occupied molecular orbital (HOMO, left) and the lowest unoccupied molecular orbital (LUMO, right) of C1 by VMD program [S22]. The structure of the ground state of C1 was optimized at B3LYP/6-31G(d) level of theory in EtOH medium.

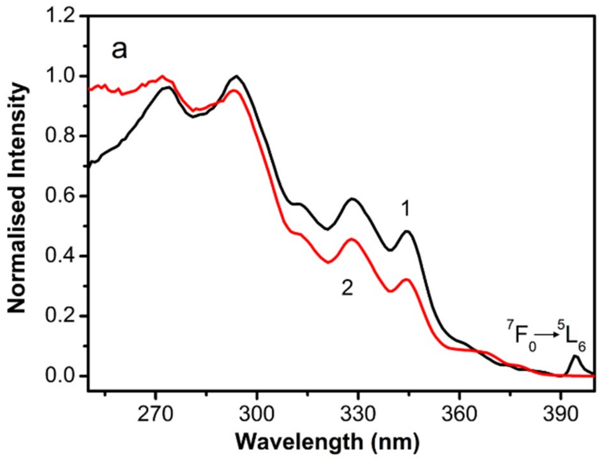

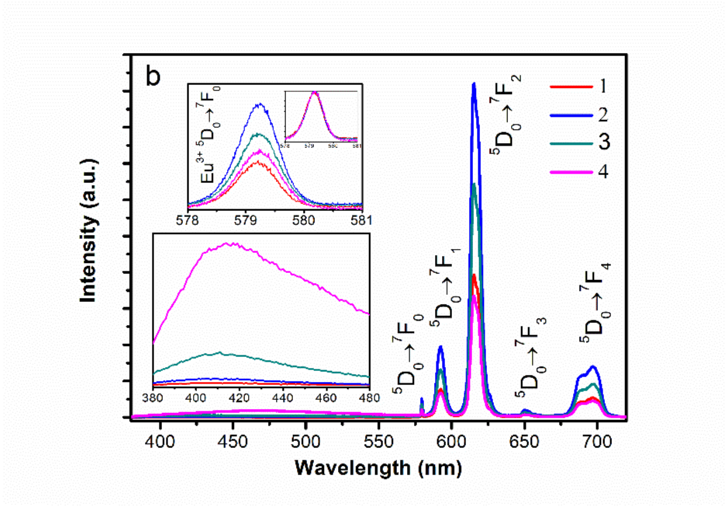

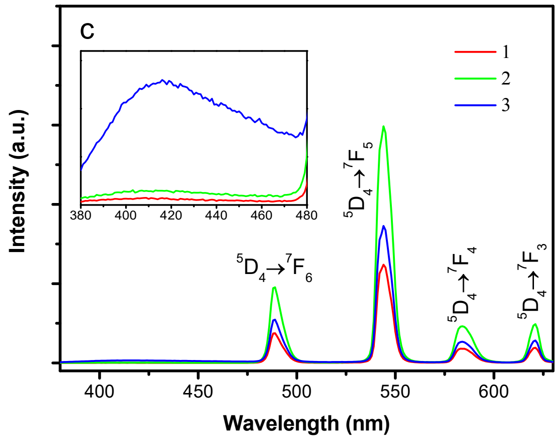

Figure S6. (a) Excitation spectra of 1) Eu-I and 2) Tb-I monitored at 615 and 545 nm, respectively; (b) emission spectra of Eu-I excited at 1) 275, 2) 295, 3) 310 and 4) 350 nm; (c) emission spectra of Tb-I excited at 1) 275, 2) 295 and 3) 350 nm.

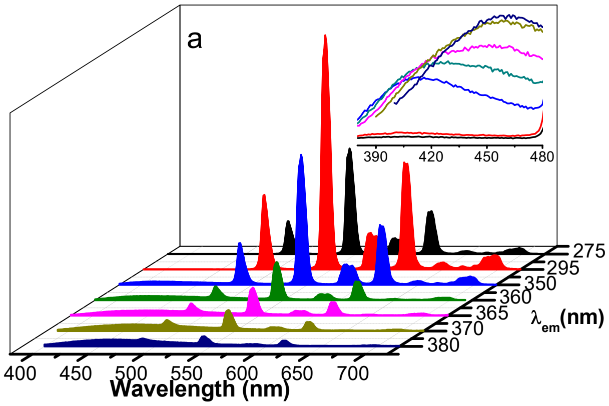

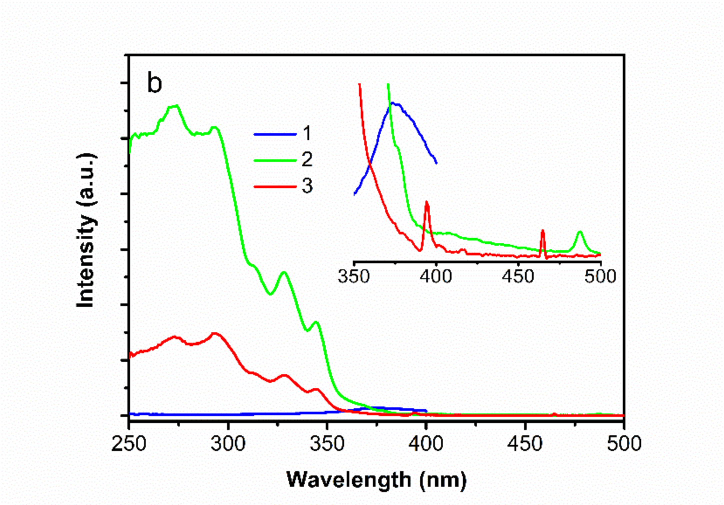

Figure S7. (a) Emission spectra of EuTb-I excited at 295, 350, 365, 370 and 380 nm; Inset shows the magnification part of wavelength region of 400 to 475 nm, (b) excitation spectra of EuTb-I monitored at 1) 460, 2) 544 and 3) 615 nm; Inset shows the magnification part of wavelength region of 350 to 500 nm.

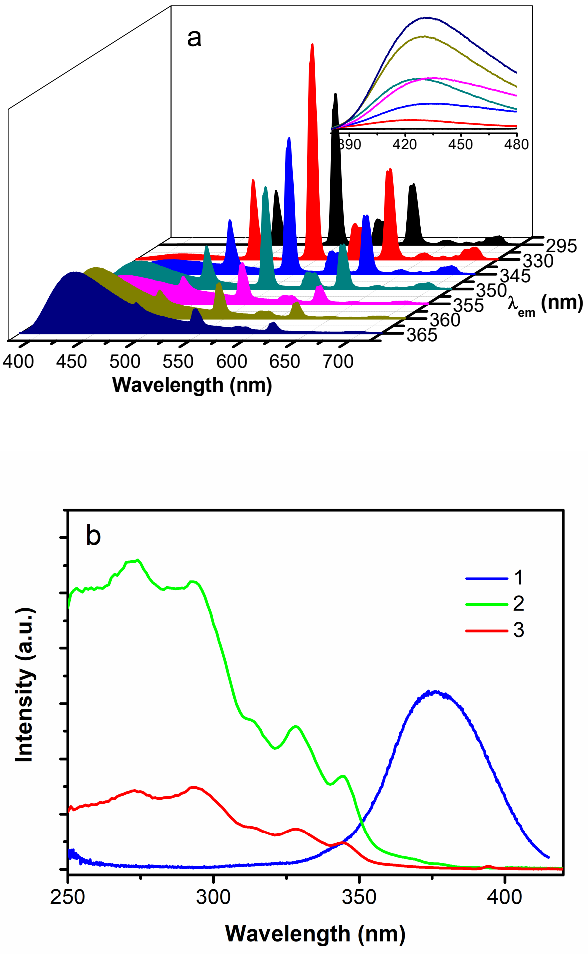

Figure S8. (a) Emission spectra of EuTbC1-I excited at different wavelength from 295 to 365 nm; Inset shows the magnification part of wavelength region of 400 to 480 nm. (b) excitation spectra of EuTbC1-I monitored at 1) 430, 2) 545 and 3) 615 nm.

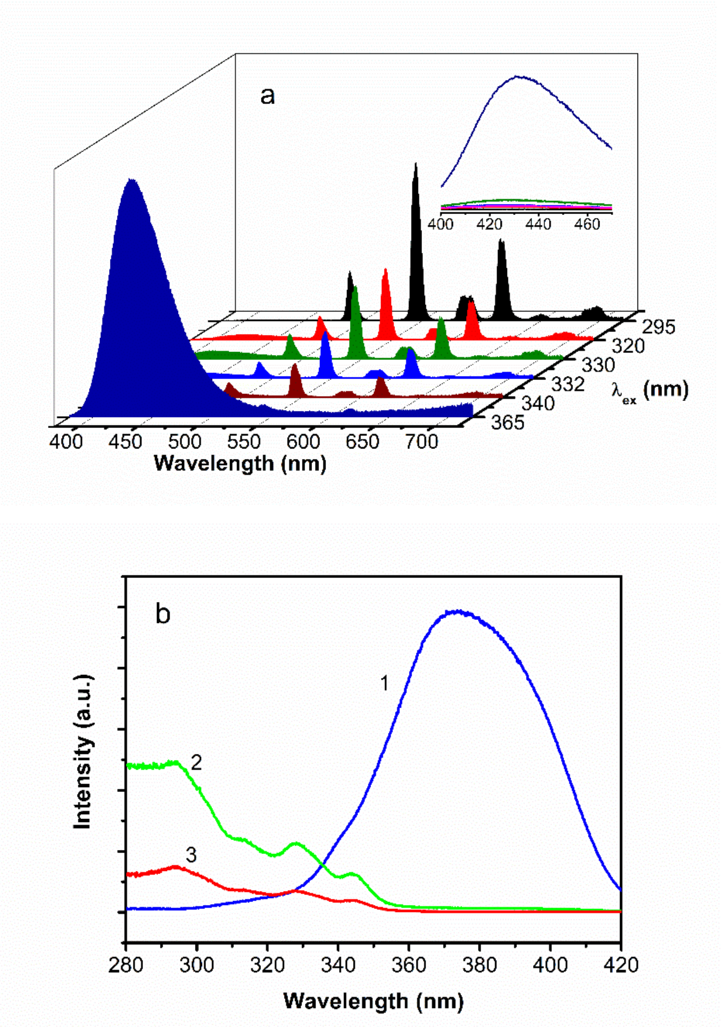

Figure S9. (a) Emission spectra of EuTbC1-II excited at different wavelength from 275 to 365 nm; Inset shows the magnification part of wavelength region of 400 to 470 nm. (b) excitation spectra of EuTbC1-II monitored at 1) 430, 2) 545 and 3) 615 nm.

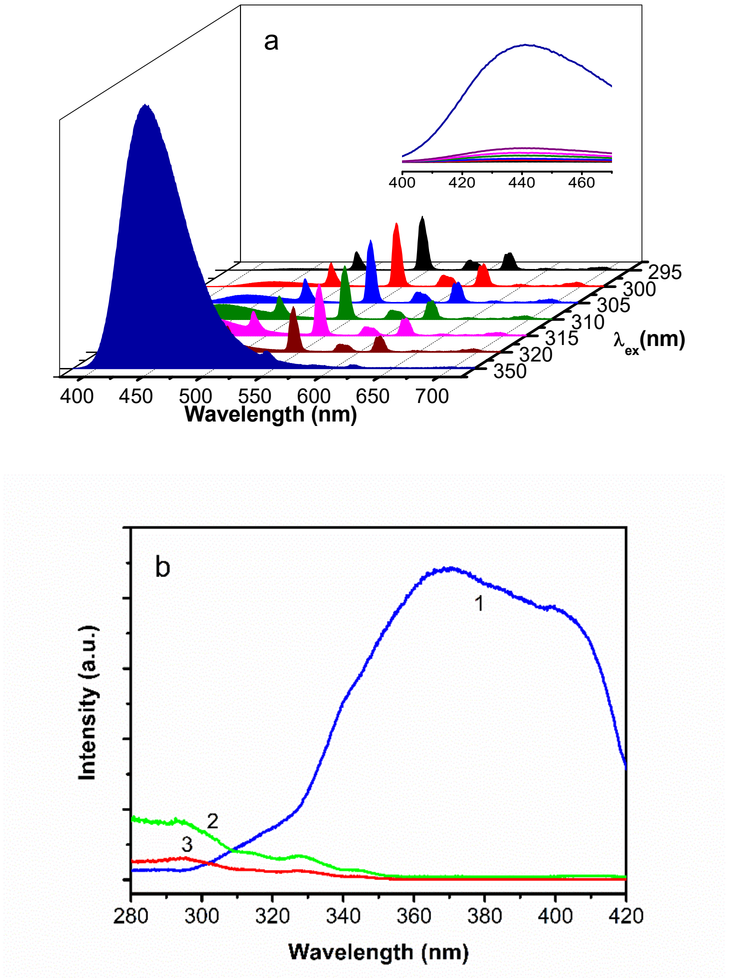

Figure S10. (a) Emission spectra of EuTbC1-III excited at different wavelength from 295 to 350 nm; Inset shows the magnification part of wavelength region of 400 to 470 nm. (b) excitation spectra of EuTbC1-III monitored at 1) 440, 2) 545 and 3) 615 nm.

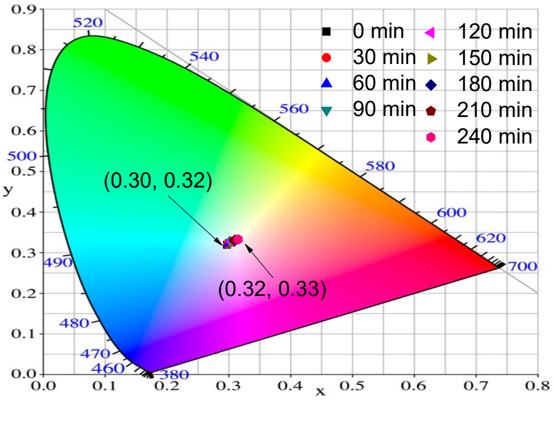

Figure S11. CIE chromaticity color diagram showing the EuTbC1-I emission color coordinates (300 K) at different irradiation times.

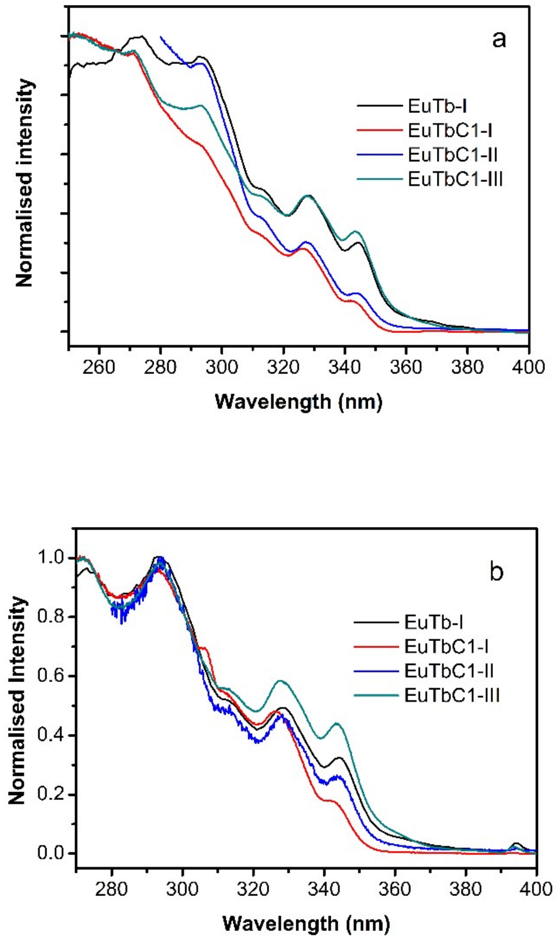

Figure S12. Excitation spectra (270–400 nm) of EuTb-I, EuTbC1-I, EuTbC1-II and EuTbC1-III monitored at (a) 545 nm and (b) 615 nm.

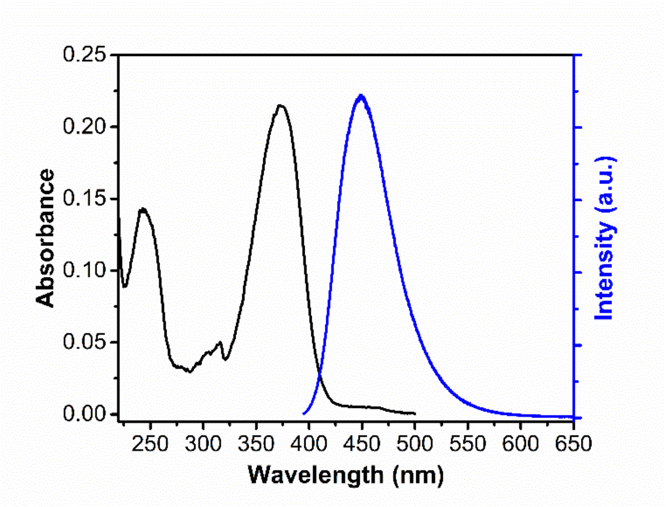

Figure S13. UV–Vis absorption (left) and emission (right, λ_{ex} = 373 nm) spectra of C1 in EtOH (1.0×10^{-5} M).

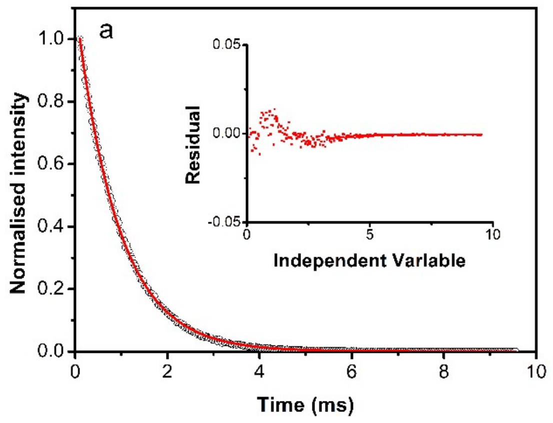

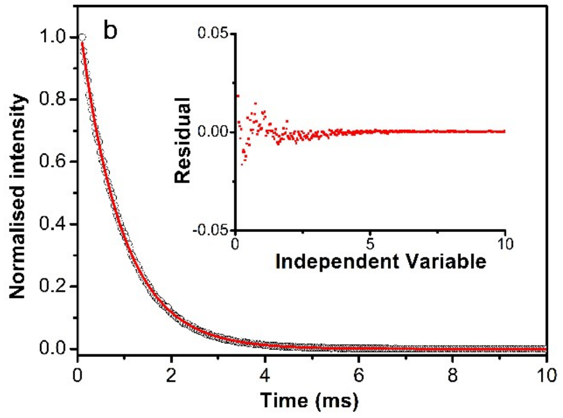

Figure S14. Emission decay curves of (a) Tb-I and (b) EuTb-I, both excited at 295 nm and monitored at 545 nm. The solid lines correspond to the data best fir (r>0.9) using a single exponential function; the fit regular residual plots are shown as insets.

The energy transfer efficiency (η_{ET}) from Tb^{3+} ions to Eu^{3+} ions was calculated using $\eta_{ET} = 1- (\tau/\tau_0)$ [S23,S24] to be 2.6%, here, τ and τ_0 stand for $^5D_4$ lifetimes of Tb^{3+} ions in the presence and absence of Eu^{3+} ions, respectively.

References

S1. de Zea Bermudez, V.; Carlos, L.D.; Duarte, M.C.; Silva, M.M.; Silva, C.J.R.; Smith, M.J.; Assunção, M.; Alcácer, L. A novel class of luminescent polymers obtained by the sol–gel approach. *J. Alloys Compd.* **1998**, *275*, 21–26.

S2. Rao, X.T.; Huang, Q.; Yang, X.L.; Cui, Y.J.; Yang, Y.; Wu, C.D.; Chen, B.L.; Qian, G.D. Color tunable and white light emitting Tb^{3+} and Eu^{3+} doped lanthanide metal−organic framework materials. *J. Mater. Chem.* **2012**, *22*, 3210−3214.

S3. Sava, D.F.; Rohwer, L.E.S.; Rodriguez, M.A.; Nenoff, T.M. Intrinsic broad-band white-light emission by a tunned, corrugated metal-organic framework. *J. Am. Chem. Soc.* **2012**, *134*, 3983−3986.

S4. Ma, M.L.; Qin, J.H.; Ji, C.; Xu, H.; Wang, R.; Li, B.J.; Zang, S.Q.; Hou, H.W.; Batten, S.R. Anionic porous metal-organic framework with novel 5-connected *vbk* topology for rapid absorption of dyes and tunable white light emission. *J. Mater. Chem. C* **2014**, *2*, 1085−1093.

S5. Sun, C.Y.; Wang, X.-L.; Zhang, X.; Qin, C.; Li, P.; Su, Z.M.; Zhu, D.X.; Shan, G.G.; Shao, K.Z.; Wu, H.; Li, J. Efficient and tunable white-light emission of metal-organic frameworks by iridium-complex encapsulation. *Nat. Commun.* **2013**, *4*, 2717.

S6. Xie, W.; Qin, J.S.; He, W.W.; Shao, K.Z.; Su, Z.M.; Du, D.Y.; Li, S.L.; Lan, Y.Q. A metal-organic framework composite for efficient white-light emission by iridium-complex encapsulation. *Inorg. Chem. Front.* **2017**, *4*, 547−552.

S7. Huo, R.; Li, X.; and Ma, D. Lanthanide coordination frameworks constructed from 1,3-benzenedicarboxylate, oxalate and 1,10-phenanthroline: crystal structure, multicolor luminescence and high-efficiency white light emission, *CrysEngComm*, **2015**, *17*, 3838−3844.

S8. Song, S.; Li, X.; Zhang, Y.H. Huo, R.; and Ma, D. White light emission by a lanthanide doped Sm(III) framework constructed from 4-sulfobenzoate and 1H-imidazo[4,5-f][1,10]-phenanthroline, *Dalton Trans.* **2014**, *43*, 5974−5977.

S9. Liu, Z.F; Wu, M.F.; Wang, S.H.; Zheng, F.K.; Wang, G.E.; Chen, J.; Xiao, Y.; Wu, A.Q.; Guo, G.C.; and Huang, J.S. Eu^{3+}-doped Tb^{3+} metal-organic frameworks emitting tunable three primary colors towards white light, *J. Mater. Chem. C* **2013**, *1*, 4634−4639.

S10. Zhang, F.M.; Yan, P.F.; Li, H.F.; Zou, X.Y.; Hou, G.F.; and Li, G.M. Towards full-color-tunable emission of two component Eu(III)-doped Gd(III) coordination frameworks by the variation of excitation light, *Dalton Trans.* **2014**, *43*, 12574–12581.

S11. Yang, Q.Y.; Wu, K.; Jiang, J.J.; Hsu, C.W.; Pan, M.; Lehn, J.M.; Su, C.Y. Pure white-light and yellow-to-blue emission tuning in single crystals of Dy(III) metal-organic frameworks, *Chem. Commun.* **2014**, *50*, 7702−7704.

S12. Song, T.; Zhang, G.G.; Cui, Y.J.; Yang, Y.; and Qian, G.D. Encapsulation of coumarin dye within lanthanide MOFs as highly efficient white-light-emitting phosphors for white LEDs, *CrystEngComm* **2016**, *18*, 8366−8371.

S13. Cui, Y.J.; Song, T.; Yu, J.C.; Yang, Y.; Wang, Z.Y.; Qian, G.D. Dye encapsulated metal-organic framework for warm-white LED with high color-rendering index, *Adv. Funct. Mater.* **2015**, *25*, 4796−4802.

S14. Wen, Y.H.; Sheng, T.L.; Zhu, X.Q.; Zhuo, C.; Li, H.R.; Hu, S.M.; Zhu, Q.L.; and Wu, X.T. Introduction of red-green-blue fluorescent dyes into a metal-organic framework for tunable white light emission, *Adv. Mater.* **2017**, *29*, 1700778.

S15. Yang, J.H.; Yan, Y.; Hui, Y.H.; and Huang, J.B. White emission thin films based on rationally designed supramolecular coordination polymers, *J. Mater. Chem. C* **2017**, *5*, 5083−5089.

S16. Chen, W.; Fan, R.Q.; Zhang, H.J.; Dong, Y.W.; Wang, P.; and Yang, Y.L. Tunable white-light emission

PMMA-supported film materials containing lanthanide coordination polymers: preparation, characterization, and properties, *Dalton Trans.* **2017**, *46*, 4265–4277.

S17. Sutar, P.; Suresh, V.M.; and Maji, T.K. Tunable emission in lanthanide coordination polymer gels based on a rationally designed blue emissive gelator, *Chem. Commun.* **2015**, *51*, 9876–9879.

S18. Laishram, R.; Bhowmik, S.; and Maitra, U. White light emitting soft materials from off-the-shelf ingredients, *J. Mater. Chem. C* **2015**, *3*, 5885−5889.

S19. Song, S.; Li, X.; Zhang, Y.H. White light emission of an Eu(III)-doped Gd(III) complex with 3-sulfobenzoate and 1H-imidazo[4,5-f][1,10]-phenanthroline, *Dalton Trans.* **2013**, *42*, 10409−10412.

S20. Zhang, H.B.; Shan, X.C.; Zhou, L.J.; Lin, P.; Li, R.F.; Ma, E.; Guo, X.G.; and Du, S.W. Full-color fluorescent materials based on mixed-lanthanide(III) metal-organic complexes with high-efficiency white light emission, *J. Mater. Chem. C* **2013**, *1*, 888−891.

S21. Frisch, M.J.; Trucks, G.W.; Schlegel, H.B.; Scuseria, G.E.; Robb, M.A.; Cheeseman, J.R.; Scalmani, G.; Barone, V.; Mennucci, B.; Petersson, G.A.; Nakatsuji, H.; Caricato, M.; Li, X.; Hratchian, H.P.; Izmaylov, A.F.; Bloino, J.; Zheng, G.; Sonnenberg, J.L.; Hada, M.; Ehara, M.; Toyota, K.; Fukuda, R.; Hasegawa, J.; Ishida, M.; Nakajima, T.; Honda, Y.; Kitao, O.; Nakai, H.; Vreven, T.; J. Montgomery, Jr., J.A.; Peralta, J.E.; Ogliaro, F.; Bearpark, M.; Heyd, J.J.; Brothers, E.; Kudin, K.N.; Staroverov, V.N.; Keith, T.; Kobayashi, R.; Normand, J.; Raghavachari, K.; Rendell, A.; Burant, J.C.; Iyengar, S.S.; Tomasi, J.; Cossi, M.; Rega, N.; Millam, J.M.; Klene, M.; Knox, J.E.; Cross, J.B.; Bakken, V.; Adamo, C.; Jaramillo, J.; Gomperts, R.; Stratmann, R.E.; Yazyev, O.; Austin, A.J.; Cammi, R.; Pomelli, C.; Ochterski, J.W.; Martin, R.L.; Morokuma, K.; Zakrzewski, V.G.; Voth, G.A.; Salvador, P.; Dannenberg, J.J.; Dapprich, S.; Daniels, A.D.;. Farkas, Ö.; Foresman, J.B.; Ortiz, J.V.; Cioslowski, J.; Fox, D.J.; Gaussian 09, Rev. B.01, Gaussian. *Inc., Wallingford CT* **2009.**

S22. Humphrey, W.; Dalke, A.; Schulten, K. VMD-Visual molecular dynamics. *J. Molec. Graphics* **1996**, *14*, 33–38.

S23. Rodrigues, M.O. Dutra, J.D.L.; Nunes, L.A.O.; de Sa, G.F.; de Azevedo, W.M.; Silva, P.; Paz, F.A.A.; Freire, R.O.; and Junior, S.A. *J. Phys. Chem. C* **2012**, *116*, 19951–19957.

S24. Piguet, C.; Bünzli, J.C.G.; Bernardinelli, G.; Hopfgartner, G.; and Williams, A.F. *J. Am. Chem. Soc.* **1993**, *115*, 8197–8206.

© 2018 by the authors. Submitted for possible open access publication under the terms and conditions of the Creative Commons Attribution (CC BY) license (http://creativecommons.org/licenses/by/4.0/).
